# Set2 Methyltransferase Facilitates DNA Replication and Promotes Genotoxic Stress Responses through MBF-Dependent Transcription

**DOI:** 10.1016/j.celrep.2017.08.058

**Published:** 2017-09-12

**Authors:** Chen-Chun Pai, Anastasiya Kishkevich, Rachel S. Deegan, Andrea Keszthelyi, Lisa Folkes, Stephen E. Kearsey, Nagore De León, Ignacio Soriano, Robertus Antonius Maria de Bruin, Antony M. Carr, Timothy C. Humphrey

**Affiliations:** 1 CRUK-MRC Oxford Institute for Radiation Oncology, Department of Oncology, University of Oxford, ORCRB, Roosevelt Drive, Oxford OX3 7DQ, UK; 2 MRC Laboratory for Molecular Cell Biology, University College London, Gower Street, London WC1E 6B, UK; 3 Genome Damage and Stability Centre, School of Life Sciences, University of Sussex, Falmer, Brighton, Sussex BN1 9RQ, UK; 4 Department of Zoology, University of Oxford, South Parks Road, Oxford OX1 3PS, UK

**Keywords:** histone methylation, histone H3K36 methylation, DNA replication, MBF, *Schizosaccharomyces pombe*, Set2, ribonucleotide reductase, dNTP

## Abstract

Chromatin modification through histone H3 lysine 36 methylation by the SETD2 tumor suppressor plays a key role in maintaining genome stability. Here, we describe a role for Set2-dependent H3K36 methylation in facilitating DNA replication and the transcriptional responses to both replication stress and DNA damage through promoting *Mlu*I cell-cycle box (MCB) binding factor (MBF)-complex-dependent transcription in fission yeast. Set2 loss leads to reduced MBF-dependent ribonucleotide reductase (RNR) expression, reduced deoxyribonucleoside triphosphate (dNTP) synthesis, altered replication origin firing, and a checkpoint-dependent S-phase delay. Accordingly, prolonged S phase in the absence of Set2 is suppressed by increasing dNTP synthesis. Furthermore, H3K36 is di- and tri-methylated at these MBF gene promoters, and Set2 loss leads to reduced MBF binding and transcription in response to genotoxic stress. Together, these findings provide new insights into how H3K36 methylation facilitates DNA replication and promotes genotoxic stress responses in fission yeast.

## Highlights

• Set2 methyltransferase is required for efficient DNA replication
• Set2 loss reduces dNTP synthesis and alters replication origin firing
• Set2 promotes efficient MBF-dependent transcription
• Increasing dNTP synthesis restores replication following Set2 loss

## Graphical Abstract

**Figure:**
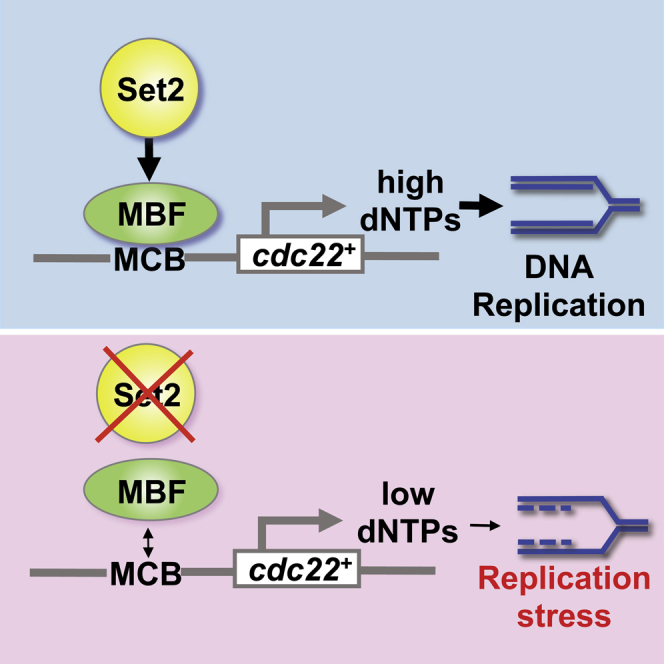

## Teaser

Pai et al. find that the Set2 methyltransferase facilitates dNTP synthesis and DNA replication through promoting MBF-dependent transcription in fission yeast. Set2 loss results in reduced ribonucleotide reductase expression, reduced dNTP synthesis, altered replication origin firing, and checkpoint-dependent S-phase delay. These findings suggest how H3K36 methylation suppresses replication stress.

## Introduction

DNA replication is a highly regulated process, and its fidelity plays a primary role in maintaining genome stability (Aguilera and Gómez-González, 2008). DNA synthesis can be divided into the stages of pre-replication complex (pre-RC) formation, replication initiation, elongation, and termination. Licensing of replication origins begins with chromatin binding of the origin recognition complex (ORC) and Cdc6/Cdc18. These factors facilitate the Cdt1-dependent loading of the mini-chromosome maintenance (MCM) complex, and subsequently multiple additional replication factors associate with the replication origin following activation of cyclin-dependent kinase (CDK) and Dbf4-dependent kinase (DDK). Pol ε is recruited to origins as part of the initiation complex and effects leading strand synthesis following helicase activation and priming by Pol α, whereas Pol δ is thought to replicate the lagging strand (Larrea et al., 2010, Pursell et al., 2007, Tanaka and Araki, 2013).

Efficient and accurate DNA replication elongation requires a balanced supply of deoxyribonucleoside triphosphates (dNTPs), generated in eukaryotes through the reduction of ribonucleoside diphosphates (NDPs) to deoxyribonucleoside diphosphates (dNDPs), which is catalyzed by ribonucleotide reductase (RNR). RNR is a heterotetrameric or possibly higher-order complex (Mathews, 2016) that in the fission yeast *Schizosaccharomyces pombe* is minimally formed from two catalytic Cdc22 subunits and two small Suc22 regulatory subunits.

RNR activity is highly regulated through a number of mechanisms (Guarino et al., 2014). In *S. pombe*, RNR is transcriptionally regulated by the *Mlu*I cell-cycle box (MCB) binding factor (MBF) complex (Liu et al., 2005). The MBF complex consists of essential core subunits including the product of the *START* gene *cdc10*^*+*^, together with Res1 and Res2, which constitute a heterodimeric DNA binding domain, and the co-activators Rep1 and Rep2 (Aves et al., 1985, Caligiuri and Beach, 1993, Miyamoto et al., 1994, Nakashima et al., 1995, Sugiyama et al., 1994, Tanaka et al., 1992, Zhu et al., 1997). The MBF complex, which is functionally analogous to the E2F complex in humans induces transcription of a set of genes, including *cdc22*^+^ (encoding the catalytic RNR subunit) *cdt1*^+^ and *cdc18*^+^ (encoding the replication licensing factors), at the G1/S transition of the cell cycle to facilitate DNA synthesis. In addition, RNR is subject to MBF-dependent transcriptional regulation by DNA integrity checkpoints in response to DNA damage and replication stresses. Following replication stress caused by RNR inhibition by hydroxyurea (HU), MBF-dependent transcription of the *cdc22*^+^ gene is activated by the S-phase checkpoint kinase Cds1 through phosphorylation of Cdc10 (Dutta et al., 2008) and by inhibition of the MBF-associated transcriptional co-repressors Nrm1 and Yox1, thus facilitating resumption of DNA replication (Caetano et al., 2011, de Bruin et al., 2006, de Bruin et al., 2008, Gómez-Escoda et al., 2011). Activation of the DNA damage checkpoint displays a more complex regulation of Cdc10-dependent transcription. Although DNA damage in G2 cells significantly upregulates a number of *Mlu*I-box transcripts, such as *cdc22*^+^, in a Rad3 and Chk1-dependent manner (Watson et al., 2004), it is clear that Chk1 activation can also inhibit MBF-dependent transcription of certain genes and phosphorylation of Cdc10 can result in its release from MBF targets (Ivanova et al., 2013). RNR activity is also post-translationally regulated through direct inhibition by Spd1 both during the cell cycle and following stress conditions (Håkansson et al., 2006, Liu et al., 2003, Moss et al., 2010). Moreover, RNR is also under allosteric control by dNTPs and NTPs. A mutation in the R1 subunit of RNR *cdc22-D57N* alleviates allosteric feedback inhibition by dATP and causes elevated dNTP pools (Caras and Martin, 1988, Chabes et al., 2003, Fleck et al., 2013).

DNA replication origin usage can be positively or negatively regulated by the chromatin environment (Aparicio et al., 2004, Knott et al., 2009, Méchali et al., 2013, Yoshida et al., 2014). Previous studies have described links between Set2-dependent histone H3 lysine 36 (H3K36) methylation and the timing of DNA replication (Biswas et al., 2008, Pryde et al., 2009), although the mechanism is unclear. Furthermore, loss of the tumor suppressor SETD2, in human cells, is associated with slower replication fork progression and with DNA replication stress (Kanu et al., 2015, Pfister et al., 2015, Shoaib and Sørensen, 2015). Moreover, studies in both fission yeast and humans showed histone H3K36 methylation to be cell-cycle regulated, with H3K36me3 levels peaking at the G1-S transition (Li et al., 2013, Pai et al., 2014). Together, these findings led us to study the function of histone H3K36 methylation in DNA replication.

Here, we establish a role for Set2 in efficient DNA replication and in facilitating efficient dNTP synthesis through promoting MBF gene transcription under normal conditions and in response to genotoxic stress.

## Results

### *set2*Δ Cells Show Perturbed Progression through S Phase

**Figure 1:**
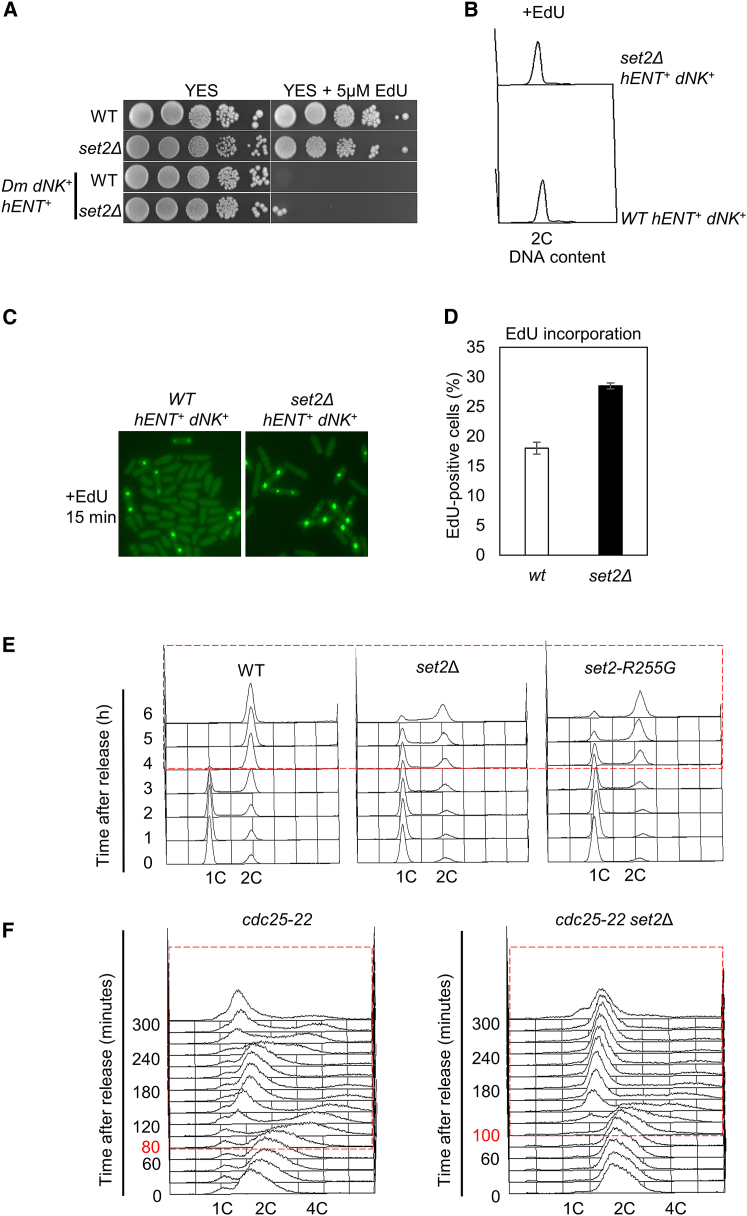
Set2 Is Required for Efficient DNA Replication (A) Effects of EdU incorporation on viability. Serial dilutions (10-fold) of wild-type, *set2*Δ, *tk hENT* (*ura4::adh-dmNK-NAT-adh-hENT1 ura4-aim*), and *tk hENT set2*Δ (*ura4::adh-dmNK-NAT-adh-hENT1 ura4-aim set2*Δ) cells were spotted on to YE3S plates containing EdU at the indicated concentration. (B) Effects of EdU incorporation on cell-cycle progression. Cells were grown in EMM containing 100 μM EdU for 15 min, and then processed and analyzed by flow cytometry. (C) Cells from (B) were fixed in 70% ethanol and conjugated to Alexa Fluor 488 azide before being imaged by fluorescence microscopy. (D) Quantification of Edu-positive cells in wild-type compared to *set2*Δ cells. (E) Wild-type, *set2*Δ or *set2-R255G* cells were arrested in G1 by nitrogen starvation and released, and samples were taken at time points indicated and subjected to FACS analysis. The red dashed line box indicates the delayed S-phase progression in *set2*Δ or *set2-R255G* compared to wild-type cells. (F) *cdc25-22* and *cdc25-22 set2*Δ cells arrested in G2 at 35.5°C for 4.25 hr followed by a shift back to the permissive temperature of 25°C. Aliquots of cells were taken every 20 min and fixed in 70% ethanol for FACS and septation index analysis (see also Figures S1B and S1C). The red dashed line box indicates the delayed S-phase progression in *cdc25-22 set2*Δ (S phase starts at 80 min after G2/M block and release) compared to *cdc25-22* cells (S phase starts at 100 min after G2/M block and release).

We wanted to investigate a possible role for Set2 in DNA replication. Both wild-type and *set2*Δ cells were grown to log phase in YES medium and processed for flow cytometry analysis. Asynchronous *set2*Δ cells do not exhibit significant S-phase delay compared to wild-type cells (Figure S1A). This result was determined by flow cytometry analysis, which may not be sufficiently sensitive to detect subtle effects on the length of S phase. We therefore performed nucleotide incorporation experiments in wild-type and *set2*Δ cells in asynchronous cultures. Efficient incorporation of 5-ethynyl-2′-deoxyuridine (EdU) into fission yeast requires expression of both thymidine kinase (TK) and a nucleoside transporter (hENT1) (Hua and Kearsey, 2011). Here, we showed that wild-type cells, or *set2*Δ cells expressing *Drosophila melanogaster* deoxyribonucleoside kinase (DmdNK) under the control of the fission yeast a*dh* promoter, together with the human equilibrative nucleoside transporter (hENT1) (*adh-Dm-dNK-adh-hENT1*) (Fleck et al., 2017) are sensitive to 5 μM EdU, whereas wild-type or *set2*Δ cells are not sensitive to EdU (Figure 1A), consistent with previous studies showing that EdU is toxic to incorporating strains (Hua and Kearsey, 2011). With medium containing 100 μM EdU for 15 min, no significant cell-cycle delay was observed in wild-type or *set2*Δ cells expressing *adh-Dm-dNK-adh-hENT1* (Figure 1B). However, during a short pulse, significantly more cells incorporating EdU were detected using fluorescent microscopy in the absence of Set2 (Figures 1C and 1D), suggesting a prolonged S phase in *set2*Δ cells.

To explore this further, we investigated a role for Set2 in DNA replication using a synchronous G1 block and release experiment. Both wild-type and *set2*Δ cells were synchronized in G1 by nitrogen starvation and released from the block at 32°C. Samples were then taken at the indicated time points and analyzed by flow cytometry. In wild-type (WT) cells, DNA replication started 2 hr after release from the G1 arrest and was finished by 5 hr (Figure 1E, WT). In contrast, we found that *set2*Δ cells displayed a significant delay in S-phase progression (Figure 1E, *set2*Δ), as was also observed in a methyltransferase-dead *set2-R255G* mutant cells (Figure 1E, *set2-R255G*). This indicates that methyltransferase activity of Set2 is required for efficient DNA replication. Similar conclusions were drawn from results obtained from cells synchronized using a *cdc25-22* G2-M block and release protocol (Figure 1F; Figures S1B and S1C). Collectively, these findings identify a role for Set2 in efficient DNA replication initiation and or elongation in synchronous cells.

### *set2*Δ Does Not Alter the Efficiency of Replication Origins

**Figure 2:**
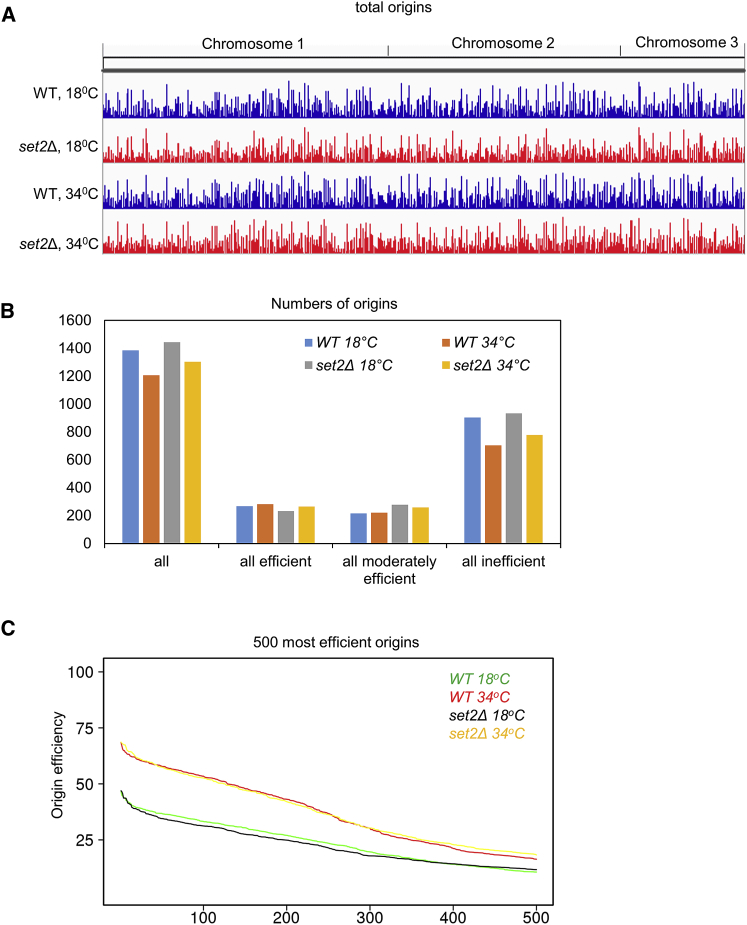
Set2 Suppresses Inefficient Origin Firing (A) The genome-wide plot of origin usage in vegetative *set2*Δ cells (red) in comparison with wild-type (blue) cells at 18°C or 34°C. Origin efficiencies were calculated from Pu-seq data. (B) The quantification of origin efficiencies in asynchronous wild-type and *set2*Δ cells at 18°C or 34°C. (C) Analysis of the 500 most efficient origins in wild-type and *set2*Δ cells at 18°C or 34°C. The x axis is the ranked 500 origins; the y axis is the efficiency from the Pu-seq data.

Given that histone modifications have been reported to affect the activity of origin firing, we examined the possible role of Set2 in regulating the pattern of replication origin usage. We performed a polymerase usage sequence (Pu-seq) method to map genome-wide origin usage as previously described (Daigaku et al., 2015, Keszthelyi et al., 2015). In wild-type cells, we identified 1,386 initiation sites at 18°C and 1,207 at 34°C including efficient (>50% usage per cell cycle), moderately efficient (50%–25%), and inefficient (>25%) origins (Figure 2A, wild-type). In the *set2*Δ background, we mapped 1,444 initiation sites at 18°C and 1,303 at 34°C (Figure 2A, *set2*Δ), suggesting a modest increase in the number of replicating origins in *set2*Δ compared to wild-type cells. Interestingly, this increase is mostly caused by increased use of inefficient origins (Figure 2B) and the overall efficiency of the majority of origins was not changed (Figure 2C)—for example, the average efficiency of the top 10% of efficient origins (Figure S2). Analysis of the distribution of origin usage in *set2*Δ cells revealed that these new inefficient origins are randomly located throughout the yeast genome. However, there is no significant difference of the number of origins identified at sub-telomeric regions of *set2*Δ compared to wild-type cells (Figure S2). In conclusion, we found that *set2*Δ subtly alters genome-wide replication origin usage but does not significantly alter the efficiency of the vast majority of origins. The main effect is to increase the total number of inefficient (previously dormant) origins used in the fission yeast genome, suggestive of a general slowdown in replication after origins have fired (Anglana et al., 2003).

### MBF-Regulated Gene Expression Is Downregulated in *set2*Δ Cells

**Figure 3:**
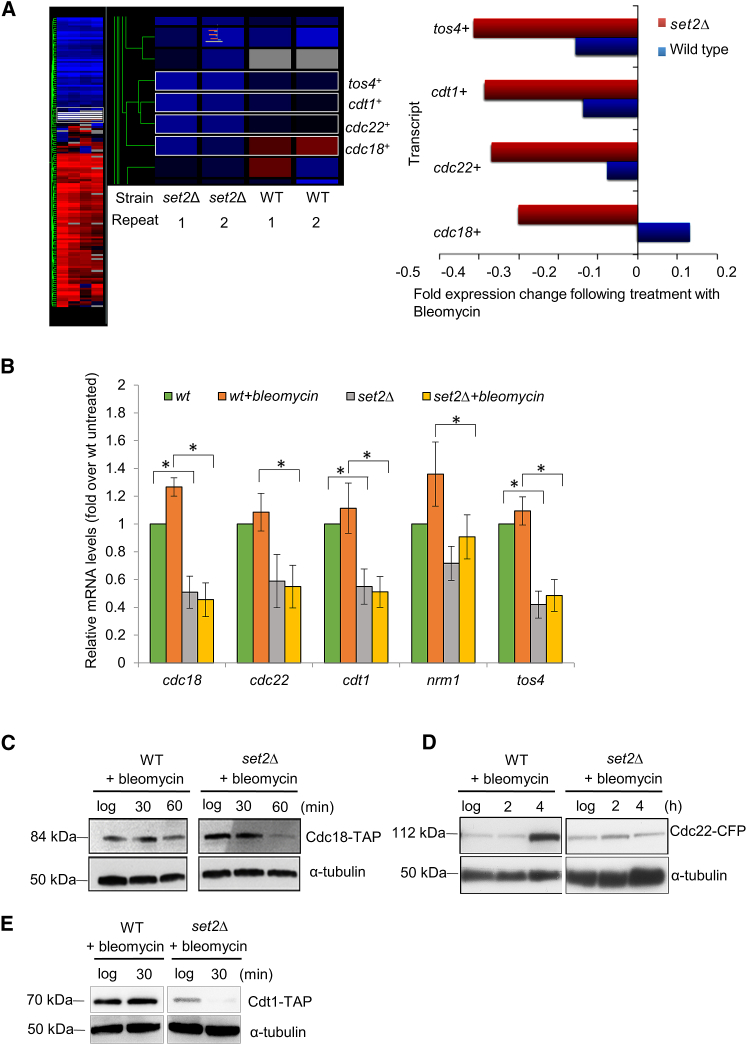
A Cluster of G1-S Genes Are Downregulated following DNA Damage in the Absence of Set2 (A) Left panel: Heatmap depicting *cdc18*^*+*^, *cdt1*^*+*^, *cdc22*^*+*^, and *tos4*^*+*^ transcript levels in wild-type and *set2*Δ cells following 30 min of treatment with 5 μg/mL bleomycin. Blue depicts downregulated compared to before damage, and red depicts upregulated compared to before damage. Right panel: Quantification of *cdc18*^*+*^, *cdt1*^*+*^, *cdc22*^*+*^, and *tos4*^*+*^ transcript levels in wild-type (blue) and *set2*Δ (red) cells following 30 min of treatment with 5 μg/mL bleomycin. Data represent mean of two experiments with independently derived RNA. (B) Quantification of *cdc18*^*+*^, *cdt1*^*+*^, *cdc22*^*+*^, *nrm1*^*+*^, and *tos4*^*+*^ transcript levels in wild-type and *set2*Δ cells following 30 min of treatment with 5 μg/mL bleomycin. Error bars represent SD from three biological replicates. The asterisk (^∗^) represents significant difference compared with wild-type and *set2*Δ (p < 0.05, t test). (C) The protein levels of Cdc18 were examined in wild-type and *set2*Δ cells following 5 μg/mL bleomycin treatment. Samples of cells were taken at the indicated time points, and cell extracts were made using TCA method. Cdc18 was detected using an antibody against the TAP tag. α-Tubulin is shown as a loading control. (D) The protein levels of Cdc22 were examined in in wild-type and *set2*Δ cells following 5 μg/mL bleomycin addition. Total cell extracts were prepared at the indicated times and analyzed by western blotting. Cdc22 was detected using an anti-GFP antibody. α-Tubulin is shown as a loading control. (E) The protein levels of Cdt1 were examined in wild-type and *set2*Δ cells following 5 μg/mL bleomycin addition. Samples of cells were taken at the indicated time points, and cell extracts were made using TCA method. Cdt1 was detected using an antibody against the TAP tag.

We have previously investigated gene expression levels in response to DNA damage in a *set2*Δ background (Pai et al., 2014). We found a number of genes to be upregulated and downregulated in response to DNA damage compared to wild-type in a *set2*Δ background. Notably, a cluster of four transcripts: *cdc18*^*+*^, *cdt1*^*+*^, *cdc22*^*+*^, and *tos4*^*+*^, involved in cell-cycle regulation, were consistently downregulated in *set2*Δ cells following treatment with bleomycin for 30 min when compared to wild-type (Figure 3A). The products of two of these genes, Cdt1 and Cdc18, are required for Mcm2-7 association with ORC in pre-RC formation (Nishitani et al., 2000). The third, Cdc22, is the large subunit of RNR whose interaction with Suc22 (the small subunit) is required for dNTP production (Fernandez Sarabia et al., 1993). The fourth, Tos4, is a putative G1-S transcription factor containing a forkhead domain, and is an effector of the DNA replication checkpoint (Bastos de Oliveira et al., 2012, Horak et al., 2002).

Analysis of the promoters of these genes indicated that they are all cell-cycle regulated and under the transcriptional control of the MBF complex, strongly suggesting that Set2 has a function in MBF transcription activation. Accordingly, following qPCR validation of the above results, we found *set2*Δ-dependent downregulation of *cdc18*^*+*^, *cdt1*^*+*^, *cdc22*^*+*^, and *tos4*^*+*^, both in the absence and presence of bleomycin, suggesting a role for Set2 in facilitating basal, as well as damage-induced MBF transcription (Figure 3B). Furthermore, we found one other MBF-dependent gene that also showed reduced transcription in *set2Δ* cells compared to wild-type following bleomycin treatment: *nrm1*^*+*^ (Figure 3B). However, other MBF-dependent genes such as *ctp1*^*+*^ and *mrc1*^*+*^ showed no alteration in transcription based on microarray analysis (data not shown). In accordance with a role for Set2 in MBF activation following DNA damage, we found that protein levels of Cdc18, Cdc22, and Cdt1 were downregulated in response to bleomycin treatment in a *set2*Δ background compared to wild-type cells (Figures 3C–3E).

### Licensing Factors Cdt1 and Cdc18 Are Misregulated in *set2*Δ Cells

**Figure 4:**
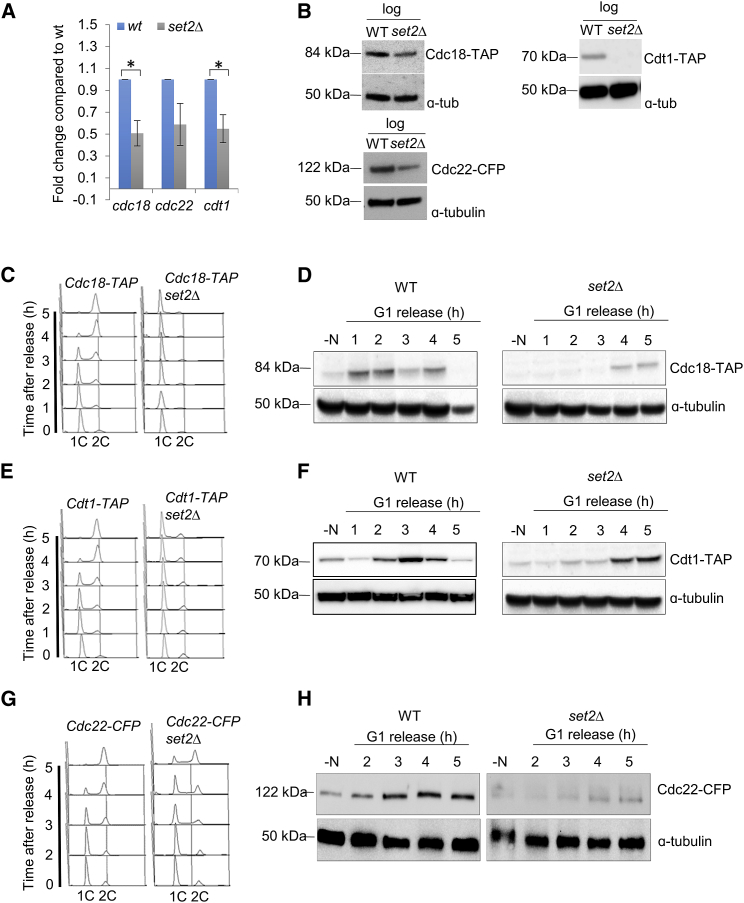
Replication Factors Are Misregulated in *set2*Δ Cells (A) Transcript levels of *cdc18*^*+*^, *cdc22*^*+*^, and *cdt1*^*+*^ were established in exponentially growing wild-type and *set2*Δ cells. RNA was extracted with Qiagen RNeasy kit and relative transcript levels of *cdc18*^*+*^, *cdc22*^*+*^, and *cdt1*^*+*^ were established by RT-qPCR. Error bars represent SD of three biological repeats. The asterisk (^∗^) represents significant difference compared with wild-type and *set2*Δ. (B) Cdc18, Cdt1, or Cdc22 protein levels of exponentially growing wild-type and *set2*Δ cells. Cell extracts were prepared from vegetative cells and processed for western blot. Immunoblots of total cell lysates were probed with PAP or GFP antibody. α-Tubulin is shown as a loading control. (C) *cdc18-TAP* or *cdc18-TAP set2*Δ cells were arrested in G1 by nitrogen starvation and released, and samples were taken at time points indicated and subjected to FACS analysis. (D) In parallel, *cdc18-TAP* and *cdc18-TAP set2*Δ cells were processed for western blotting at indicated times. (E) *cdt1-TAP* or *cdt1-TAP set2*Δ cells were arrested in G1 by nitrogen starvation, released, and samples taken at time points indicated and subjected to FACS analysis. (F) In parallel, *cdt1-TAP* and *cdt1-TAP set2*Δ cells were processed for western blotting at indicated times. (G) A similar experiment to that described in (C) was carried out using *cdc22-CFP* or *cdc22-CFP set2*Δ cells. (H) A similar experiment to that described in (D) was carried out using *cdc22-CFP* or *cdc22-CFP set2*Δ cells. Immunoblots of total cell extracts were probed with GFP antibody. α-Tubulin is shown as a loading control.

Given the replication delay seen in *set2*Δ cells and the involvement of three members of the cluster in efficient DNA replication, we analyzed mRNA and protein levels of these replication factors, Cdc18, Cdt1, and Cdc22, in the absence of genotoxic stress. In asynchronous cell cultures where most cells are in G2, we found that these replication factors were all downregulated in a *set2*Δ background compared to a wild-type (Figures 4A and 4B), further supporting a role for Set2 in DNA replication. Next, we investigated protein levels of the licensing factors Cdc18 and Cdt1 using a synchronous G1 block and release experiment. Both licensing factors showed oscillating protein levels in wild-type cells (Figures 4D and 4F, wild-type), in accordance with normal S-phase progression (Figures 4C and 4E). In contrast, *set2*Δ cells showed delayed expression of Cdc18 and Cdt1 after release from G1 arrest (Figures 4C–4F, *set2*Δ), and the final levels achieved were lower than seen in wild-type cells. Similar conclusions were drawn from results obtained from cells synchronized using a *cdc25-22* G2-M block and release protocol (Figures S3A and S3B). In accordance with protein levels, *set2*Δ cells also showed delayed mRNA expression of *cdc18*^*+*^ and *cdt1*^*+*^ after release from G1 arrest (Figures S4A and S4B), supporting the idea that changes are transcriptional, although there may also be post-translational changes. This result suggests that a delay in pre-RC formation or origin binding of initiating factors is contributing to the slow S phase observed for *set2*Δ cells (Figure 1). Consistent with this, previous studies in budding yeast showed that chromatin association of initiation factor Cdc45 was delayed in a *set2*Δ strain (Pryde et al., 2009), presumably due to delayed expression of licensing factors.

To see whether the delayed S-phase entry in *set2*Δ cells could be rescued by increasing the expression of Cdt1 and Cdc18, the genes were ectopically expressed from a *REP81X* plasmid in *cdc25-22* and *cdc25-22 set2*Δ cells. However, in a *cdc25-22 set2*Δ background, cells grown in the absence of thiamine had a similar profile to those grown in its presence, indicating overexpression of Cdc18 and Cdt1 did not suppress the replication defect observed in *set2*Δ cells (Figure S5). This suggests that other DNA replication steps were affected and account for the slow growth phenotype.

Of the cluster of four genes that have significantly decreased transcript levels in response to bleomycin in *set2*Δ cells, Tos4 has been identified in *S. cerevisiae* as a G1-S transcription factor (Horak et al., 2002). Given the replication delay seen in *set2*Δ cells and the involvement of the other three members of the cluster in efficient DNA replication, the cell-cycle progression of *tos4*Δ cells was analyzed. However, in contrast to the defective S-phase progression seen in *set2*Δ cells, a *cdc25-22 tos4*Δ double mutant did not exhibit significant delay in cell-cycle progression following G2-M block and release (Figure S6).

### Set2 Is Required for Balanced dNTP Synthesis

**Figure 5:**
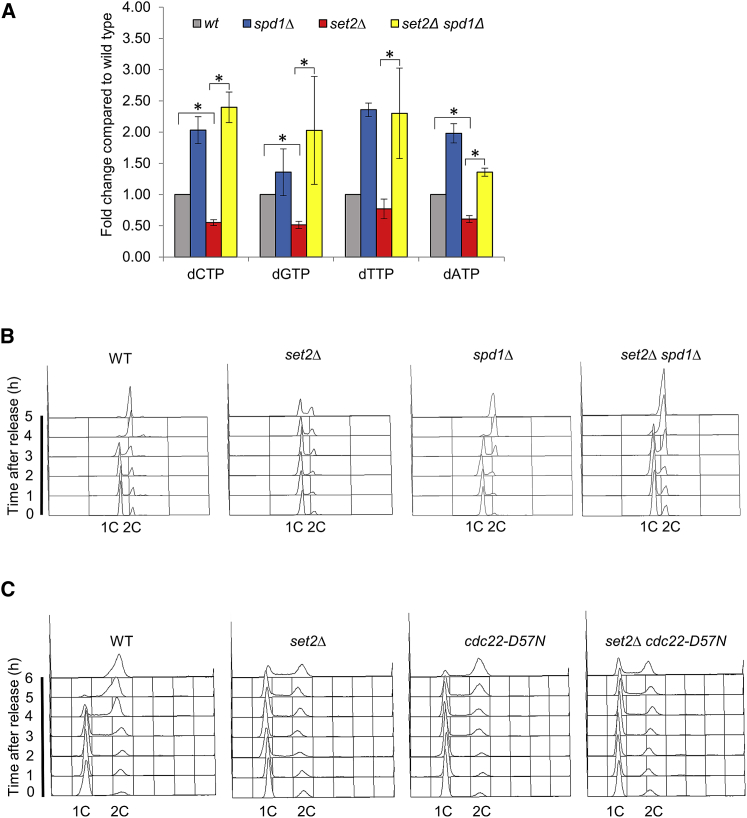
Elevated dNTP Pools Speed Up S-Phase Progression in *set2*Δ Cells (A) dNTP levels were measured in exponential growth wild-type, *spd1*Δ, *set2*Δ, and *set2*Δ *spd1*Δ strains. Means ± SEs of three experiments are shown. The asterisk (^∗^) represents significant difference compared with wild-type and *set2*Δ and *set2*Δ and *set2*Δ*spd1*Δ strains. (B) Removal of RNR inhibitor Spd1 suppresses prolong S-phase progression in *set2*Δ cells. Flow cytometry analysis of the indicated strains after release from G1 arrest into EMM+N at 32°C. (C) The S-phase failure of *set2*Δ cells was poorly suppressed by the *cdc22-D57N* mutation. Flow cytometry analysis of the indicated strains after release from G1 arrest into EMM+N at 32°C.

The four dNTP precursors for DNA synthesis are potentially limiting for initiation and elongation if levels are too low (Yekezare et al., 2013). Importantly, imbalanced dNTP pools caused by altered expression and/or activity of RNR can affect cell-cycle progression and lead to mutagenesis (Chabes and Stillman, 2007, Fleck et al., 2013). Because our earlier results showed that Cdc22 levels were also downregulated in a *set2*Δ mutant (Figure 4B), we monitored the levels of the catalytic subunit of RNR, Cdc22, in wild-type and *set2*Δ cells during a G1 block and release cell cycle. In contrast to wild-type, RNR protein and mRNA levels did not rise around the time of S phase, consistent with the slow S-phase progression seen in *set2*Δ cells (Figures 4G and 4H; Figure S4C). This result indicates that the replication defect in a *set2*Δ background could have arisen from low or imbalanced levels of dNTPs, which could possibly cause replication progression defects in a *set2*Δ background. To verify this, we measured dNTP levels in asynchronous *set2*Δ cells and found that dNTP levels were lower, with dCTP, dGTP, and dATP levels being significantly reduced (p < 0.05) compared to that of wild-type (Figure 5A), suggesting imbalanced dNTP levels could be limiting for DNA replication.

### Elevated dNTP Pools Suppress Slow Replication in *set2*Δ Cells

To confirm whether the cause of replication delay in *set2*Δ cells was through Set2-dependent dNTP synthesis during the cell cycle, we tested whether it was possible to suppress the replication defects of *set2*Δ cells by increasing dNTP pools. To achieve this, the gene encoding Spd1, an inhibitor of RNR (Liu et al., 2003, Woollard et al., 1996), was deleted in a *set2*Δ background. We found that Spd1 depletion restored dNTP levels in *set2*Δ cells (Figure 5A). We repeated the G1 block and release experiment and found that deletion of *spd1*^*+*^ considerably rescued the DNA replication delay in *set2*Δ cells (Figure 5B), consistent with a Set2 function in promoting dNTP synthesis. However, we do not exclude the possibility that Spd1 may have other functions in regulating S-phase progression in fission yeast (Fleck et al., 2017). In contrast, the S-phase failure of *set2*Δ cells was poorly suppressed by the *cdc22-D57N* mutation (Figure 5C), although the result is difficult to interpret as the single *cdc22-D57N* mutant releases poorly from the G1 block, possibly due to abnormal dNTP levels (Chabes and Stillman, 2007, Pai and Kearsey, 2017).

### S-Phase Delay in *set2*Δ Cells Is Checkpoint Dependent

**Figure 6:**
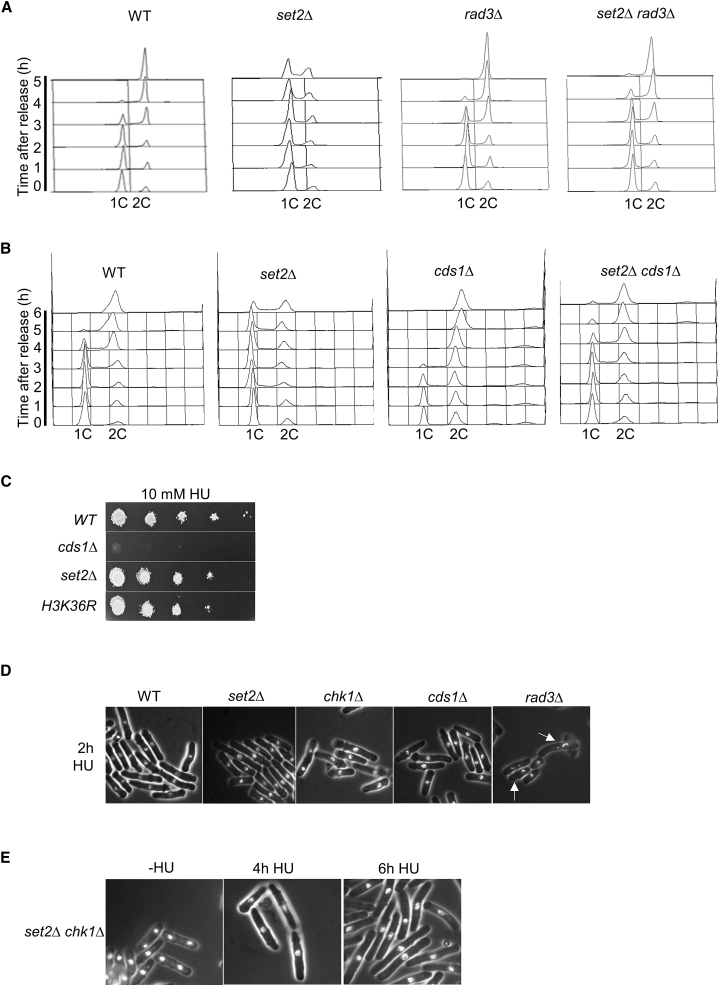
The *set2*Δ Mutation Causes a Rad3 (ATR)-Dependent S-Phase Progression Defect (A) Deletion of Rad3 (ATR) increases the speed of S-phase progression in *set2*Δ cells. Flow cytometry analysis of wild-type (WT), *set2*Δ and *set2*Δ *rad3*Δ strains after release from G1 into EMM+N at 32°C. (B) Deletion of Cds1 (Chk2) increases the speed of S-phase progression in *set2*Δ cells. Flow cytometry analysis of wild-type (WT), *set2*Δ and *set2*Δ *cds1*Δ strains after release from G1 into EMM+N at 32°C. (C) Serial dilution of a wild-type and *cds1*Δ, *set2*Δ, and *H3K36R* mutants were spotted onto YES medium containing 10 mM HU. Plates were incubated at 30°C for 2–3 days. (D) Each fixed culture from wild-type, *set2*Δ *chk1*Δ, *cds1*Δ, or *rad3*Δ strain was stained with 4′-6-diamidino-2-phenylindole (DAPI) and analyzed by fluorescence microscopy showing nuclear morphology for each strain. (E) *set2*Δ *chk1*Δ cells were grown in YES medium containing 10 mM HU at indicated times and stained with DAPI followed by microscopy analysis for nuclear morphology.

We wanted to test whether delayed S-phase progression resulting from loss of H3K36 methylation resulted directly by limiting S-phase progression through nucleotide depletion or was exacerbated by checkpoint activation resulting from dNTP depletion. Indeed, it is well established that inhibition of DNA replication due to depletion or imbalanced of dNTP pools leads to activation of the S-phase checkpoint (Enoch and Nurse, 1990, Kumar et al., 2010, Murakami and Okayama, 1995). To test this idea, we deleted *rad3*^*+*^ encoding the checkpoint sensor Rad3 (ATR) in *set2*Δ cells and monitored cell-cycle progression by fluorescent-activated cell sorting (FACS) analysis. We found that deleting *rad3*^*+*^ suppressed the prolonged S-phase progression in *set2*Δ cells (Figure 6A). Consistent with this result, deleting *cds1*^*+*^ encoding the Cds1 replication checkpoint kinase also suppressed the slow S-phase progression in *set2*Δ cells (Figure 6B). This finding suggests that imbalanced nucleotide pools activate the intra-S checkpoint resulting in S-phase delay. Alternatively, DNA synthesis from low levels of pyrimidines (dCTP or dTTP) could cause DNA damage that activates the S-phase checkpoint, therefore arresting the cell-cycle progression (Kumar et al., 2010).

### DNA Integrity Checkpoint Activation Is Proficient in *set2*Δ Cells

The above result implies that DNA replication checkpoint signaling is not affected by inactivation of Set2. To confirm this, we analyzed the function of Set2 in checkpoint signaling in response to replication stress. In contrast to deleting the checkpoint genes, *set2*Δ or *H3K36R* mutants exhibited only modest HU sensitivity (Figure 6C; Figure S7A). Treatment with HU was not found to result in an increased “cut” phenotype, in which incompletely replicated DNA is divided into two daughter cells, in a *set2*Δ background (Figure 6D), suggesting that mitosis was arrested in the presence of incomplete DNA replication and that the intra-S-phase checkpoint in *set2*Δ cells was intact. In fission yeast, Cds1 is the effector kinase of the DNA synthesis checkpoint pathway, stabilizing stalled forks and triggering transcriptional activation of G1-S-specific genes (Dutta et al., 2008). Moreover, Cds1 and Chk1 establish the redundant pathways of HU-induced checkpoint arrest and double mutants exhibit mitotic catastrophe in response to DNA damage (Lindsay et al., 1998, Zeng et al., 1998). We therefore further investigated the role of Set2 in HU-induced checkpoint arrest through examining the effect of inactivating Set2 in *chk1*Δ cells. We observed that, like wild-type cells, *set2*Δ *chk1*Δ cells exhibited an elongated phenotype in the presence of HU, confirming that Set2 is not required for proficient Cds1 activation (Figure 6E). Consistent with this result, HU-induced activation of Cds1 was not affected by the loss of Set2 (Figure S7B) as previously reported in fission yeast (Kim et al., 2008). We did not detect Cds1 phosphorylation in unperturbed *set2*Δ cells, perhaps due to low checkpoint activation reflecting a cell-cycle delay rather than an arrest. Taken together, these results suggest that Set2 is proficient in replication checkpoint activation.

Roles have recently been described for the budding yeast Set2 and the human homolog, SETD2, in DNA damage signaling (Carvalho et al., 2014, Jha and Strahl, 2014). To investigate whether Set2 acts in the DNA damage checkpoint response, the DNA damage-induced mobility shift of Chk1 on SDS-PAGE was examined. The modified form of Chk1 was detected in bleocin-treated wild-type and *set2*Δ cells, indicating that Chk1 activation is not affected by loss of Set2 (Figure S7C). Furthermore, we found that DNA damage-dependent Crb2 phosphorylation is still observed in the absence of Set2 (Figure S7C). Consistent with this result, *set2*Δ cells exhibited an elongated phenotype in the presence of bleocin in comparison with *chk1*Δ cells (Figure S7D). Taken together, these results suggested that *set2*Δ cells are proficient for the G2-M arrest in the presence of DNA damage in *S. pombe*.

### Set2 Facilitates Efficient DNA Replication through Promoting MBF-Induced dNTP Synthesis

**Figure 7:**
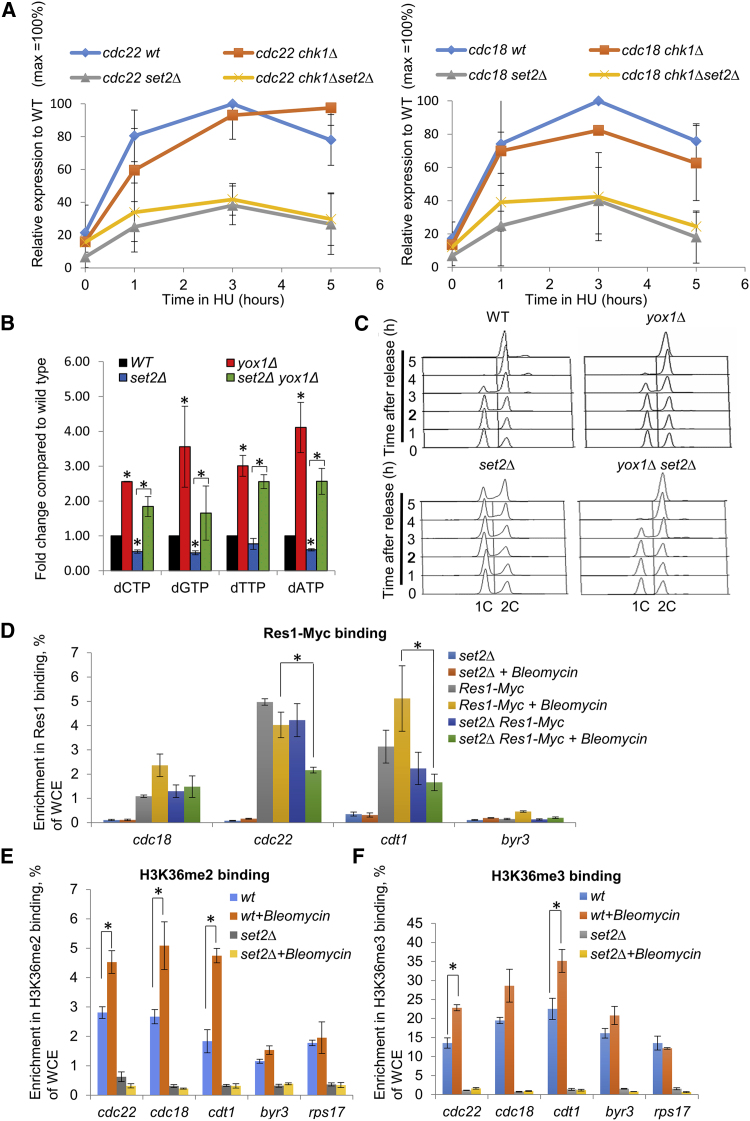
Set2 Facilitates Efficient Binding of MBF Components in Response to Genotoxic Stress (A) Set2 has a direct function in regulating MBF activity in response to replication stress. Left panel: quantification of *cdc22*^*+*^ transcript levels in wild-type, *chk1*Δ, *set2*Δ, and *set2*Δ *chk1*Δ cells following 5-hr treatment with 10 mM HU. Data represent mean of three experiments with independently derived RNA. Right panel: quantification of *cdc18*^*+*^ transcript levels in wild-type, *chk1*Δ, *set2*Δ, and *set2*Δ *chk1*Δ cells following 5-hr treatment with 10 mM HU. Data represent mean of three experiments with independently derived RNA. (B) dNTP levels were measured in exponential growth wild-type, *yox1*Δ, *set2*Δ, and *set2*Δ *yox1*Δ strains. Means ± SEs of three experiments are shown. The asterisk (^∗^) represents significant difference compared with WT and *set2Δ*, WT and *yox1Δ*, *set2*Δ and *set2*Δ*yox1*Δ. (C) Removal of MBF inhibitor Yox1 speeds up S-phase progression in *set2*Δ cells. Flow cytometry analysis of the indicated strains after release from G1 arrest into EMM+N at 32°C. (D) Levels of Res1-Myc binding at *cdc18*^*+*^, *cdc22*^*+*^, *cdt1*^*+*^, and *byr3*^*+*^ (negative control) promoters were shown in the presence or absence of DNA damage. Cells of logarithmically growing cultures (YES medium) of *set2*Δ, *res1-Myc*, *set2*Δ *res1-Myc* strains were collected for ChIP before and 30 min after treatment with 5 μg/mL bleomycin. Data represent the mean of three experiments with independently derived RNA and error bars (±SE) are shown. The asterisk (^∗^) represents significant difference compared with wild-type (p < 0.05, t test). (E) Set2-dependent di-methylation of H3K36 at promoters is induced upon genotoxic stress. Levels of H3K36me3 at *cdc18*^*+*^, *cdc22*^*+*^, *cdt1*^*+*^, *byr3*^*+*^ (negative control), and *rps17*^*+*^ (negative control) promoters were presented in wild-type or *set2*Δ strains without and with DNA damage treatment. Cells of logarithmically growing wild-type and *set2*Δ cultures were collected for ChIP before and 30 min after treatment with 5 μg/mL bleomycin. Data represent the mean of three experiments with independently derived RNA and error bars (±SE) are shown. The asterisk (^∗^) represents significant difference compared with wild-type (p < 0.05, t test). (F) Set2-dependent tri-methylation of H3K36 at promoters is induced upon genotoxic stress. Levels of H3K36me3 at *cdc18*^*+*^, *cdc22*^*+*^, *cdt1*^*+*^, *byr3*^*+*^ (negative control), and *rps17*^*+*^ (negative control) promoters were presented in wild-type or *set2*Δ strains without and with DNA damage treatment. Cells of logarithmically growing wild-type and *set2*Δ cultures were collected for ChIP before and 30 min after treatment with 5 μg/mL bleomycin. Data represent the mean of three experiments with independently derived RNA and error bars (±SE) are shown. The asterisk (^∗^) represents significant difference compared with wild-type (p < 0.05, t test).

Our results suggest that Set2 is required for induced expression of a subset of MBF-dependent genes in response to bleomycin treatment. Previous studies have shown that MBF-dependent transcription is induced in response to replication stress (Bertoli et al., 2013), but can be both induced and inhibited in response to DNA damage (Ivanova et al., 2013, Watson et al., 2004). Therefore, Set2 could either have a role in the induction of MBF-dependent transcription in response to replication stress or the prevention of DNA damage-induced inhibition of MBF target gene expression. To test this, we first performed RT-qPCR analysis of MBF-dependent genes following HU treatment to assess the role of Set2 in replication stress-induced transcription. Cells were treated with HU for an extended period of time to establish whether Set2 is required for the induction and/or maintenance of MBF-dependent gene expression in response to replication stress. We found that Set2 is required for rapid induction and sustained expression of MBF-dependent *cdc22*^+^ or *cdc18*^+^ under HU-induced replication stress conditions (Figures 7A and 7B).

To examine the possibility that Set2 depletion causes a higher degree of replication stress-induced DNA damage, which in turn indirectly activates Chk1 in response to replication stress and inactivates MBF at *cdc22*^*+*^ and *cdc18*^*+*^ promoters, we analyzed these MBF targets in *set2*Δ *chk1*Δ compared to *set2*Δ cells. The *set2*Δ and *set2*Δ *chk1*Δ cells show a similar pattern of MBF-dependent expression (Figure 7A), indicating the decrease in MBF-dependent transcription in HU is likely to be a direct effect of *set2*^*+*^ deletion, rather than DNA damage-dependent inhibition. This result supports the idea that the loss of G1-S transcription in a *set2*Δ background was due to MBF complex dysfunction. In support of our findings, deletion of *yox1*^+^, encoding the MBF transcription repressor Yox1, significantly elevated dNTP pools in *set2*Δ cells (Figure 7B), and further suppressed the prolonged-S phase in *set2*Δ cells, presumably due to elevated RNR levels (Figure 7C). These results together support a role for Set2-dependent H3K36 methylation in facilitating efficient DNA synthesis through regulation of MBF-dependent gene expression, primarily through regulation of RNR transcript levels.

To further investigate a role for Set2 in promoting MBF-dependent transcriptional regulation, chromatin immunoprecipitation (ChIP) analysis of MBF binding was assessed in wild-type and *set2*Δ cells. We found that binding of the MBF transcription factor subunit Res1 at *cdc22*^*+*^ and *cdt1*^*+*^ promoters is significantly reduced in a *set2*Δ background compared to wild-type in the presence of bleomycin (Figure 7D). Furthermore, we investigated whether Set2 was recruited to these gene promoters in a wild-type background following genotoxic stress. ChIP analysis indicated that the promoter regions of these MBF-dependent genes underwent H3K36 di- and tri-methylation (Figures 7E and 7F), demonstrating that Set2-dependent H3K36 methylation has the potential to regulate the binding of MBF. Together, these results suggest a role for Set2 in facilitating recruitment of the MBF transcription factor to MCB-containing promoters to facilitate their expression in response to genotoxic stress.

## Discussion

We have established important roles for Set2-dependent H3K36 methylation in facilitating efficient DNA replication, together with promoting the transcriptional responses to both replication and genotoxic stresses. Our data support a role for H3K36 methylation in facilitating these functions through promoting the basal and genotoxic stress-induced transcription of a subset of MBF-regulated genes.

We find Set2 facilitates efficient DNA replication through maintaining dNTP pools. Accordingly, the S-phase delay observed in a *set2*Δ background can be suppressed by increasing dNTP levels either through deleting the MBF repressor gene *yox1*^*+*^, or by deletion of *spd1*^*+*^ encoding an inhibitor of RNR in a *set2*Δ background. These data support an important role for Set2 in facilitating efficient DNA replication through MBF-dependent dNTP synthesis. Although replication origins fire with largely equivalent efficiency in wild-type and *set2*Δ cells, we observed that a number of additional low-efficiency origins (dormant origins) are utilized in *set2*Δ cells compared to wild-type cells. We found the S-phase delay in a *set2*Δ background could be largely suppressed by deleting Rad3 (ATR) or Cds1 (CHK2), strongly suggesting that the S-phase delay arose through activation of the intra-S checkpoint in a *set2*Δ background. These data are consistent with an insufficient supply of dNTPs in a *set2*Δ background leading to increased replication stress and or DNA damage, and to subsequent checkpoint-dependent S-phase delay.

Analysis of DNA replication origins in a *set2*Δ background revealed an increase in the number of inefficient origins being fired, which are randomly distributed throughout the yeast genome as would be expected when replication is slowed (see above). Interestingly, a role for Set2 has recently been described in facilitating the recruitment of Shugoshin (Sgo2) to sub-telomeric regions and for the formation of highly condensed chromatin bodies or “knobs” flanking telomeric heterochromatin (Matsuda et al., 2015). Sgo2 regulates replication timing at the sub-telomeres by limiting Sld3 loading, and sub-telomeric regions were found to replicate early in *sgo2*Δ cells (Tashiro et al., 2016). Therefore, loss of Set2 could regulate early firing of sub-telomeric origins through facilitating increased Sld3 loading.

Our findings support a role for Set2 in facilitating the transcription of a subset of MBF-regulated genes, both at the basal level (without damage) and when activated in response to genotoxic stress. Consistent with this, Set2 loss resulted in significantly reduced mRNA and protein expression of a subset of MBF genes including *cdc18*^*+*^, *cdt1*^*+*^, and *cdc22*^*+*^ in asynchronous or synchronized conditions or in response to HU or bleomycin. How might Set2 facilitate MBF-dependent transcription? Our data are consistent with a role for Set2-dependent H3K36 methylation in promoting MBF-dependent transcription through regulating the binding and or activity of the MBF complex to the promoters of MBF target genes. In this respect, we find significantly reduced levels of Res1 at MBF promoters in a *set2*Δ background following DNA damage. We note that genes whose expression is particularly sensitive to loss of Set2 exhibit a promoter pattern associated with two MCB elements (5′-AACGCG-3′ and 5′-CGCGNCGCG-3′), which is present only in a subset of MBF-regulated transcripts. Therefore, Set2 loss may affect expression of genes whose promoters contain similar MCB arrangements more robustly. However, the precise mechanism as to how H3K36 methylation promotes MBF binding to target genes in the absence or presence of genotoxic stress is currently unclear and will require further investigation. We previously showed that H3K36 modification was cell-cycle regulated with H3K36 tri-methylation being associated with an increased 1C DNA content (Pai et al., 2014). Such timing would be consistent with a role in facilitating MBF-dependent transcription, described here.

The findings presented here strongly support a highly conserved role for H3K36 methylation in facilitating efficient DNA replication from yeast to mammalian cells in response to replication stress (Kanu et al., 2015, Pfister et al., 2015). As loss of SETD2 is observed in a number of human cancer types (Li et al., 2016), delineating the role of H3K36 methylation in cellular stress responses in both yeast and human cells will be of particular biological and clinical interest.

## Experimental Procedures

### Yeast Strains, Media, and Genetic Methods

The strains used in this study are listed in Table S1. Standard media and growth conditions were used throughout this work. Cultures were grown in rich media (YE6S) or Edinburgh minimal media (EMM) at 32°C with shaking, unless otherwise stated. Nitrogen starvation was carried out using EMM lacking NH_4_Cl.

### Serial Dilution Assay

A dilution series for the indicated mutant cells was spotted onto YES plates. Plates were incubated at 25°C, 32°C, or 36°C for 2–3 days before analysis.

### Cell-Cycle Analysis

Cells were synchronized in G1 by nitrogen starvation. Log-phase cells were released into minimal medium lacking nitrogen for 16 hr. During the time course of nitrogen re-feeding, samples of cells were taken for methanol/acetone fixation, followed by microscopy and FACS analysis. Cells were synchronized in G2-M by Cdc25 inactivation. *cdc25-22* cells were grown at 25°C, shifted to 35.5°C for 4.25 hr, and then released, and samples were taken for FACS analysis at indicated time points.

### Microarray Analysis

Microarray analysis was performed as previously described (Pai et al., 2014, Rallis et al., 2013). In brief, Alexa 555- or 647-labeled cDNA was produced from the RNA, using a Superscript direct cDNA labeling system (Invitrogen) and Alexa 555 and 647 dUTP label mix. The cDNA was then purified using an Invitrogen PureLink PCR Purification system. The cDNA was hybridized to the array using a Gene Expression Hybridization kit (Agilent). The array was an Agilent custom-designed array containing 60-mer oligonucleotides synthesized in situ on the array and contained 4 × 44,000 probes. Following hybridization for at least 17 hr, the array was washed using a Gene Expression Wash Buffer kit (Agilent) and scanned in an Agilent Array Scanner. The microarray signal was extracted using GenePix.

### Microscopy Analysis

Asynchronous cell culture was treated with 10 mM HU at indicated temperature before being fixed in methanol. Samples were rehydrated and stained with 4′,6-diamidino-2-phenylindole (DAPI) before examination using Zeiss Axioplan 2ie microscope, Hamamatsu Orca ER camera, and micromanager software. For visualization of Rad22-GFP foci, cells were incubated at 25°C or 35°C for 5 hr before being fixed and visualized as above.

### Pu-Seq

Pu-seq technique was performed as previously described (Daigaku et al., 2015). Briefly, DNA was extracted from cells grown to log phase either at 18°C or 34°C as indicated. For “wild-type” datasets, two strains were used, both strains containing *rnh201* deletion together with either polymerase δ (*cdc6-L591G*) or polymerase ε (*cdc20-M630F*) mutations. These strains incorporate more rNTPs on the strands synthetized by the mutant polymerase. These sites can be mapped by Pu-seq. For the *set2*Δ datasets, the strain also contained these mutations along with *rnh201* and *cdc6-L591G* or *cdc20-M630F*. The isolated DNA was then subjected to alkali treatment (0.3 M NaOH, 2 hr at 55°C), which digested the DNA at the positions of rNTP incorporation and also separated the double-stranded DNA. The resulting ssDNA fragments were size selected on agarose gel (fragments between 300 and 500 bp were isolated). These fragments were then used for creating strand-specific next-generation sequencing libraries and sequenced on a Next-seq Illumina platform resulting in ∼10M reads from each strain. Reads were aligned to the *Schizosaccharomyces pombe* reference sequence (http://www.pombase.org/downloads/genome-datasets), the reads were mapped using bowtie2 and the data were analyzed and origin positions and efficiencies were determined using the tools published and described in detail in Daigaku et al. (2015) and Keszthelyi et al. (2015) with default variables except that the “percentile threshold for origins” option was set to 0.2 = 20th percentile. Efficient origins were determined as origins with higher than 50% efficiency and inefficient origins had less than 25% efficiency. Because our previous work has indicated subtle changes to origin firing at different temperatures, we performed the analysis on cultures grown at either 18°C or 34°C.

### ChIP

Yeast culture was grown in YES to OD_595_ = 0.3–0.5. 45 mL of culture was incubated with 1% of formaldehyde for 20 min for cross-linking. The reaction was quenched with 125 mM glycine. Cells were lysed with lysis buffer (50 mM HEPEs-KOH, pH 7.5, 140 mM sodium chloride, 1% Triton X-100, 0.1% sodium deoxycholate, 1 mM EDTA) supplemented with protease inhibitors (cOmplete Tablets Mini, EASYpack; Roche) by vortexing with glass beads (0.5 mm; Biospec Products). Chromatin was sheared to 500–1,000 bp by sonication (amplitude, 100%; process time, 5 min; ON time, 30 s; OFF time, 2 min). Chromatin lysate was incubated with specific antibodies (anti-H3K36me2 Active Motif 39255; antiH3K36me3 Active Motif 61101; Myc-tag Santa Cruz 9E10: sc-40) at 4°C overnight rotating. Chromatin was pulled down with cold 50% protein A-Sepharose beads in lysis buffer (Sigma; P3391) for 3 hr and washed six times with washing buffer (50 mM Tris-HCl, 1% Triton X-100, 150 mM sodium chloride, 5 mM EDTA, 0.5% NP-40). De-cross-linking was performed with 10% Chelex in ultra-pure water and boiling for 10 min. DNA enrichment was established by qPCR with SYBR green according to the manufacturer’s recommendations and percentage of the whole-cell extract method. Primers used in this study are listed in Table S2.

### Protein Analysis

Protein extracts were made by trichloroacetic acid (TCA) extraction and analyzed by western blotting as described previously (Pai et al., 2014). TAP-tagged proteins were detected with peroxidase-antiperoxidase-soluble complex (P1291; Sigma). Cdc22-GFP was detected using antibody 1181446000 (Roche), and α-tubulin was detected with antibody T5168 (Sigma). Phos-tag Acrylamide gel was used to detect Cds1-P (Wako).

### HPLC Analysis of dNTP

dNTP analysis was carried out as previously described (Moss et al., 2010).

### qPCR Analysis

qPCR analysis was carried out as previously described (Caetano et al., 2014). Total RNA was prepared using the RNeasy Plus Kit (Qiagen) as indicated in the manufacturer’s manual. Transcript levels were determined by RT-qPCR using the iScript One-Step RT-PCR kit with SYBR Green Supermix (Bio-Rad). RT-PCRs were run on a Chromo-4 Real-Time PCR Detector (Bio-Rad) and obtained experimental values analyzed using MJ Opticon Analysis Software 3.0. Furthermore, data were normalized against actin and investigated using the C(t) method. Primers used in this study are listed in Table S2.

## Author Contributions

C.-C.P., A.K., R.S.D., A.K., L.F., N.D.L., I.S., and S.E.K. designed and performed the experiments. C.-C.P., R.S.D., and T.C.H. designed experiments and analyzed data. C.-C.P. and T.C.H. wrote the manuscript with input from all authors. Experiments in Figure 1 were performed by C.-C.P., R.S.D., and S.E.K.; in Figure 2 were performed by A.K. and A.M.C.; in Figure 3 were performed by C.-C.P., A.K., and R.A.M.d.B.; in Figure 4 were performed by C.-C.P., A.K., and N.D.L.; in Figure 5 were performed by C.-C.P., L.F., and N.D.L.; in Figure 6 were performed by C.-C.P. and N.D.L.; in Figure 7 were performed by C.-C.P., A.K., and R.A.M.d.B.; and in Figure S7B were performed by I.S.

## Supplemental Information

Document S1. Figures S1–S7 and Tables S1 and S2

Document S2. Article plus Supplemental Information

## References

[bib1] Aguilera A., Gómez-González B. (2008). Genome instability: a mechanistic view of its causes and consequences. Nat. Rev. Genet..

[bib2] Anglana M., Apiou F., Bensimon A., Debatisse M. (2003). Dynamics of DNA replication in mammalian somatic cells: nucleotide pool modulates origin choice and interorigin spacing. Cell.

[bib3] Aparicio J.G., Viggiani C.J., Gibson D.G., Aparicio O.M. (2004). The Rpd3-Sin3 histone deacetylase regulates replication timing and enables intra-S origin control in *Saccharomyces cerevisiae*. Mol. Cell. Biol..

[bib4] Aves S.J., Durkacz B.W., Carr A., Nurse P. (1985). Cloning, sequencing and transcriptional control of the *Schizosaccharomyces pombe* cdc10 “start” gene. EMBO J..

[bib5] Bastos de Oliveira F.M., Harris M.R., Brazauskas P., de Bruin R.A., Smolka M.B. (2012). Linking DNA replication checkpoint to MBF cell-cycle transcription reveals a distinct class of G1/S genes. EMBO J..

[bib6] Bertoli C., Skotheim J.M., de Bruin R.A. (2013). Control of cell cycle transcription during G1 and S phases. Nat. Rev. Mol. Cell Biol..

[bib7] Biswas D., Takahata S., Xin H., Dutta-Biswas R., Yu Y., Formosa T., Stillman D.J. (2008). A role for Chd1 and Set2 in negatively regulating DNA replication in *Saccharomyces cerevisiae*. Genetics.

[bib8] Caetano C., Klier S., de Bruin R.A. (2011). Phosphorylation of the MBF repressor Yox1p by the DNA replication checkpoint keeps the G1/S cell-cycle transcriptional program active. PLoS One.

[bib9] Caetano C., Limbo O., Farmer S., Klier S., Dovey C., Russell P., de Bruin R.A. (2014). Tolerance of deregulated G1/S transcription depends on critical G1/S regulon genes to prevent catastrophic genome instability. Cell Rep..

[bib10] Caligiuri M., Beach D. (1993). Sct1 functions in partnership with Cdc10 in a transcription complex that activates cell cycle START and inhibits differentiation. Cell.

[bib11] Caras I.W., Martin D.W. (1988). Molecular cloning of the cDNA for a mutant mouse ribonucleotide reductase M1 that produces a dominant mutator phenotype in mammalian cells. Mol. Cell. Biol..

[bib12] Carvalho S., Vítor A.C., Sridhara S.C., Martins F.B., Raposo A.C., Desterro J.M.P., Ferreira J., de Almeida S.F. (2014). SETD2 is required for DNA double-strand break repair and activation of the p53-mediated checkpoint. eLife.

[bib13] Chabes A., Stillman B. (2007). Constitutively high dNTP concentration inhibits cell cycle progression and the DNA damage checkpoint in yeast *Saccharomyces cerevisiae*. Proc. Natl. Acad. Sci. USA.

[bib14] Chabes A., Georgieva B., Domkin V., Zhao X., Rothstein R., Thelander L. (2003). Survival of DNA damage in yeast directly depends on increased dNTP levels allowed by relaxed feedback inhibition of ribonucleotide reductase. Cell.

[bib15] Daigaku Y., Keszthelyi A., Müller C.A., Miyabe I., Brooks T., Retkute R., Hubank M., Nieduszyski C.A., Carr A.M. (2015). A global profile of replicative polymerase usage. Nat. Struct. Mol. Biol..

[bib16] de Bruin R.A., Kalashnikova T.I., Chahwan C., McDonald W.H., Wohlschlegel J., Yates J., Russell P., Wittenberg C. (2006). Constraining G1-specific transcription to late G1 phase: the MBF-associated corepressor Nrm1 acts via negative feedback. Mol. Cell.

[bib17] de Bruin R.A., Kalashnikova T.I., Aslanian A., Wohlschlegel J., Chahwan C., Yates J.R., Russell P., Wittenberg C. (2008). DNA replication checkpoint promotes G1-S transcription by inactivating the MBF repressor Nrm1. Proc. Natl. Acad. Sci. USA.

[bib18] Dutta C., Patel P.K., Rosebrock A., Oliva A., Leatherwood J., Rhind N. (2008). The DNA replication checkpoint directly regulates MBF-dependent G1/S transcription. Mol. Cell. Biol..

[bib19] Enoch T., Nurse P. (1990). Mutation of fission yeast cell cycle control genes abolishes dependence of mitosis on DNA replication. Cell.

[bib20] Fernandez Sarabia M.J., McInerny C., Harris P., Gordon C., Fantes P. (1993). The cell cycle genes *cdc22*^*+*^ and *suc22*^*+*^ of the fission yeast *Schizosaccharomyces pombe* encode the large and small subunits of ribonucleotide reductase. Mol. Gen. Genet..

[bib21] Fleck O., Vejrup-Hansen R., Watson A., Carr A.M., Nielsen O., Holmberg C. (2013). Spd1 accumulation causes genome instability independently of ribonucleotide reductase activity but functions to protect the genome when deoxynucleotide pools are elevated. J. Cell Sci..

[bib22] Fleck O., Fahnøe U., Løvschal K.V., Gasasira M.U., Marinova I.N., Kragelund B.B., Carr A.M., Hartsuiker E., Holmberg C., Nielsen O. (2017). Deoxynucleoside salvage in fission yeast allows rescue of ribonucleotide reductase deficiency but not Spd1-mediated inhibition of replication. Genes (Basel).

[bib23] Gómez-Escoda B., Ivanova T., Calvo I.A., Alves-Rodrigues I., Hidalgo E., Ayté J. (2011). Yox1 links MBF-dependent transcription to completion of DNA synthesis. EMBO Rep..

[bib24] Guarino E., Salguero I., Kearsey S.E. (2014). Cellular regulation of ribonucleotide reductase in eukaryotes. Semin. Cell Dev. Biol..

[bib25] Håkansson P., Dahl L., Chilkova O., Domkin V., Thelander L. (2006). The *Schizosaccharomyces pombe* replication inhibitor Spd1 regulates ribonucleotide reductase activity and dNTPs by binding to the large Cdc22 subunit. J. Biol. Chem..

[bib26] Horak C.E., Luscombe N.M., Qian J., Bertone P., Piccirrillo S., Gerstein M., Snyder M. (2002). Complex transcriptional circuitry at the G1/S transition in *Saccharomyces cerevisiae*. Genes Dev..

[bib27] Hua H., Kearsey S.E. (2011). Monitoring DNA replication in fission yeast by incorporation of 5-ethynyl-2′-deoxyuridine. Nucleic Acids Res..

[bib28] Ivanova T., Alves-Rodrigues I., Gómez-Escoda B., Dutta C., DeCaprio J.A., Rhind N., Hidalgo E., Ayté J. (2013). The DNA damage and the DNA replication checkpoints converge at the MBF transcription factor. Mol. Biol. Cell.

[bib29] Jha D.K., Strahl B.D. (2014). An RNA polymerase II-coupled function for histone H3K36 methylation in checkpoint activation and DSB repair. Nat. Commun..

[bib30] Kanu N., Grönroos E., Martinez P., Burrell R.A., Yi Goh X., Bartkova J., Maya-Mendoza A., Mistrík M., Rowan A.J., Patel H. (2015). SETD2 loss-of-function promotes renal cancer branched evolution through replication stress and impaired DNA repair. Oncogene.

[bib31] Keszthelyi A., Daigaku Y., Ptasińska K., Miyabe I., Carr A.M. (2015). Mapping ribonucleotides in genomic DNA and exploring replication dynamics by polymerase usage sequencing (Pu-seq). Nat. Protoc..

[bib32] Kim H.S., Rhee D.K., Jang Y.K. (2008). Methylations of histone H3 lysine 9 and lysine 36 are functionally linked to DNA replication checkpoint control in fission yeast. Biochem. Biophys. Res. Commun..

[bib33] Knott S.R., Viggiani C.J., Tavaré S., Aparicio O.M. (2009). Genome-wide replication profiles indicate an expansive role for Rpd3L in regulating replication initiation timing or efficiency, and reveal genomic loci of Rpd3 function in *Saccharomyces cerevisiae*. Genes Dev..

[bib34] Kumar D., Viberg J., Nilsson A.K., Chabes A. (2010). Highly mutagenic and severely imbalanced dNTP pools can escape detection by the S-phase checkpoint. Nucleic Acids Res..

[bib35] Larrea A.A., Lujan S.A., Nick McElhinny S.A., Mieczkowski P.A., Resnick M.A., Gordenin D.A., Kunkel T.A. (2010). Genome-wide model for the normal eukaryotic DNA replication fork. Proc. Natl. Acad. Sci. USA.

[bib36] Li F., Mao G., Tong D., Huang J., Gu L., Yang W., Li G.-M. (2013). The histone mark H3K36me3 regulates human DNA mismatch repair through its interaction with MutSα. Cell.

[bib37] Li J., Duns G., Westers H., Sijmons R., van den Berg A., Kok K. (2016). SETD2: an epigenetic modifier with tumor suppressor functionality. Oncotarget.

[bib38] Lindsay H.D., Griffiths D.J.F., Edwards R.J., Christensen P.U., Murray J.M., Osman F., Walworth N., Carr A.M. (1998). S-phase-specific activation of Cds1 kinase defines a subpathway of the checkpoint response in *Schizosaccharomyces pombe*. Genes Dev..

[bib39] Liu C., Powell K.A., Mundt K., Wu L., Carr A.M., Caspari T. (2003). Cop9/signalosome subunits and Pcu4 regulate ribonucleotide reductase by both checkpoint-dependent and -independent mechanisms. Genes Dev..

[bib40] Liu C., Poitelea M., Watson A., Yoshida S.H., Shimoda C., Holmberg C., Nielsen O., Carr A.M. (2005). Transactivation of *Schizosaccharomyces pombe cdt2*^+^ stimulates a Pcu4-Ddb1-CSN ubiquitin ligase. EMBO J..

[bib41] Mathews C.K. (2016). The most interesting enzyme in the world. Structure.

[bib42] Matsuda A., Chikashige Y., Ding D.-Q., Ohtsuki C., Mori C., Asakawa H., Kimura H., Haraguchi T., Hiraoka Y. (2015). Highly condensed chromatins are formed adjacent to subtelomeric and decondensed silent chromatin in fission yeast. Nat. Commun..

[bib43] Méchali M., Yoshida K., Coulombe P., Pasero P. (2013). Genetic and epigenetic determinants of DNA replication origins, position and activation. Curr. Opin. Genet. Dev..

[bib44] Miyamoto M., Tanaka K., Okayama H. (1994). res2+, a new member of the cdc10+/SWI4 family, controls the “start” of mitotic and meiotic cycles in fission yeast. EMBO J..

[bib45] Moss J., Tinline-Purvis H., Walker C.A., Folkes L.K., Stratford M.R., Hayles J., Hoe K.L., Kim D.U., Park H.O., Kearsey S.E. (2010). Break-induced ATR and Ddb1-Cul4(Cdt)^2^ ubiquitin ligase-dependent nucleotide synthesis promotes homologous recombination repair in fission yeast. Genes Dev..

[bib46] Murakami H., Okayama H. (1995). A kinase from fission yeast responsible for blocking mitosis in S phase. Nature.

[bib47] Nakashima N., Tanaka K., Sturm S., Okayama H. (1995). Fission yeast Rep2 is a putative transcriptional activator subunit for the cell cycle “start” function of Res2-Cdc10. EMBO J..

[bib48] Nishitani H., Lygerou Z., Nishimoto T., Nurse P. (2000). The Cdt1 protein is required to license DNA for replication in fission yeast. Nature.

[bib49] Pai C.C., Kearsey S.E. (2017). A critical balance: dNTPs and the maintenance of genome stability. Genes (Basel).

[bib50] Pai C.C., Deegan R.S., Subramanian L., Gal C., Sarkar S., Blaikley E.J., Walker C., Hulme L., Bernhard E., Codlin S. (2014). A histone H3K36 chromatin switch coordinates DNA double-strand break repair pathway choice. Nat. Commun..

[bib51] Pfister S.X., Markkanen E., Jiang Y., Sarkar S., Woodcock M., Orlando G., Mavrommati I., Pai C.C., Zalmas L.P., Drobnitzky N. (2015). Inhibiting WEE1 selectively kills histone H3K36me3-deficient cancers by dNTP starvation. Cancer Cell.

[bib52] Pryde F., Jain D., Kerr A., Curley R., Mariotti F.R., Vogelauer M. (2009). H3 k36 methylation helps determine the timing of cdc45 association with replication origins. PLoS One.

[bib53] Pursell Z.F., Isoz I., Lundström E.B., Johansson E., Kunkel T.A. (2007). Yeast DNA polymerase epsilon participates in leading-strand DNA replication. Science.

[bib54] Rallis C., Codlin S., Bähler J. (2013). TORC1 signaling inhibition by rapamycin and caffeine affect lifespan, global gene expression, and cell proliferation of fission yeast. Aging Cell.

[bib55] Shoaib M., Sørensen C.S. (2015). Epigenetic deficiencies and replicative stress: driving cancer cells to an early grave. Cancer Cell.

[bib56] Sugiyama A., Tanaka K., Okazaki K., Nojima H., Okayama H. (1994). A zinc finger protein controls the onset of premeiotic DNA synthesis of fission yeast in a Mei2-independent cascade. EMBO J..

[bib57] Tanaka S., Araki H. (2013). Helicase activation and establishment of replication forks at chromosomal origins of replication. Cold Spring Harb. Perspect. Biol..

[bib58] Tanaka K., Okazaki K., Okazaki N., Ueda T., Sugiyama A., Nojima H., Okayama H. (1992). A new cdc gene required for S phase entry of *Schizosaccharomyces pombe* encodes a protein similar to the *cdc10*^+^ and *SWI4* gene products. EMBO J..

[bib59] Tashiro S., Handa T., Matsuda A., Ban T., Takigawa T., Miyasato K., Ishii K., Kugou K., Ohta K., Hiraoka Y. (2016). Shugoshin forms a specialized chromatin domain at subtelomeres that regulates transcription and replication timing. Nat. Commun..

[bib60] Watson A., Mata J., Bähler J., Carr A., Humphrey T. (2004). Global gene expression responses of fission yeast to ionizing radiation. Mol. Biol. Cell.

[bib61] Woollard A., Basi G., Nurse P. (1996). A novel S phase inhibitor in fission yeast. EMBO J..

[bib62] Yekezare M., Gómez-González B., Diffley J.F. (2013). Controlling DNA replication origins in response to DNA damage—inhibit globally, activate locally. J. Cell Sci..

[bib63] Yoshida K., Bacal J., Desmarais D., Padioleau I., Tsaponina O., Chabes A., Pantesco V., Dubois E., Parrinello H., Skrzypczak M. (2014). The histone deacetylases sir2 and rpd3 act on ribosomal DNA to control the replication program in budding yeast. Mol. Cell.

[bib64] Zeng Y., Forbes K.C., Wu Z., Moreno S., Piwnica-Worms H., Enoch T. (1998). Replication checkpoint requires phosphorylation of the phosphatase Cdc25 by Cds1 or Chk1. Nature.

[bib65] Zhu Y., Takeda T., Whitehall S., Peat N., Jones N. (1997). Functional characterization of the fission yeast Start-specific transcription factor Res2. EMBO J..

