# Supplementary material for: Set2 Methyltransferase Facilitates DNA Replication and Promotes Genotoxic Stress Responses through MBF-Dependent Transcription
Source: Cell Rep. 2017 Sep 12;20(11):2693–705. doi: 10.1016/j.celrep.2017.08.058 (PMC5608972; doi:10.1016/j.celrep.2017.08.058)
Supplement: Document S1. Figures S1–S7 and Tables S1 and S2 [file mmc1.pdf]

Cell Reports, Volume *20*

Supplemental Information

Set2 Methyltransferase Facilitates DNA Replication and Promotes Genotoxic Stress Responses through MBF-Dependent Transcription

Chen-Chun Pai, Anastasiya Kishkevich, Rachel S. Deegan, Andrea Keszthelyi, Lisa Folkes, Stephen E. Kearsey, Nagore De León, Ignacio Soriano, Robertus Antonius Maria de Bruin, Antony M. Carr, and Timothy C. Humphrey

Supplementary information

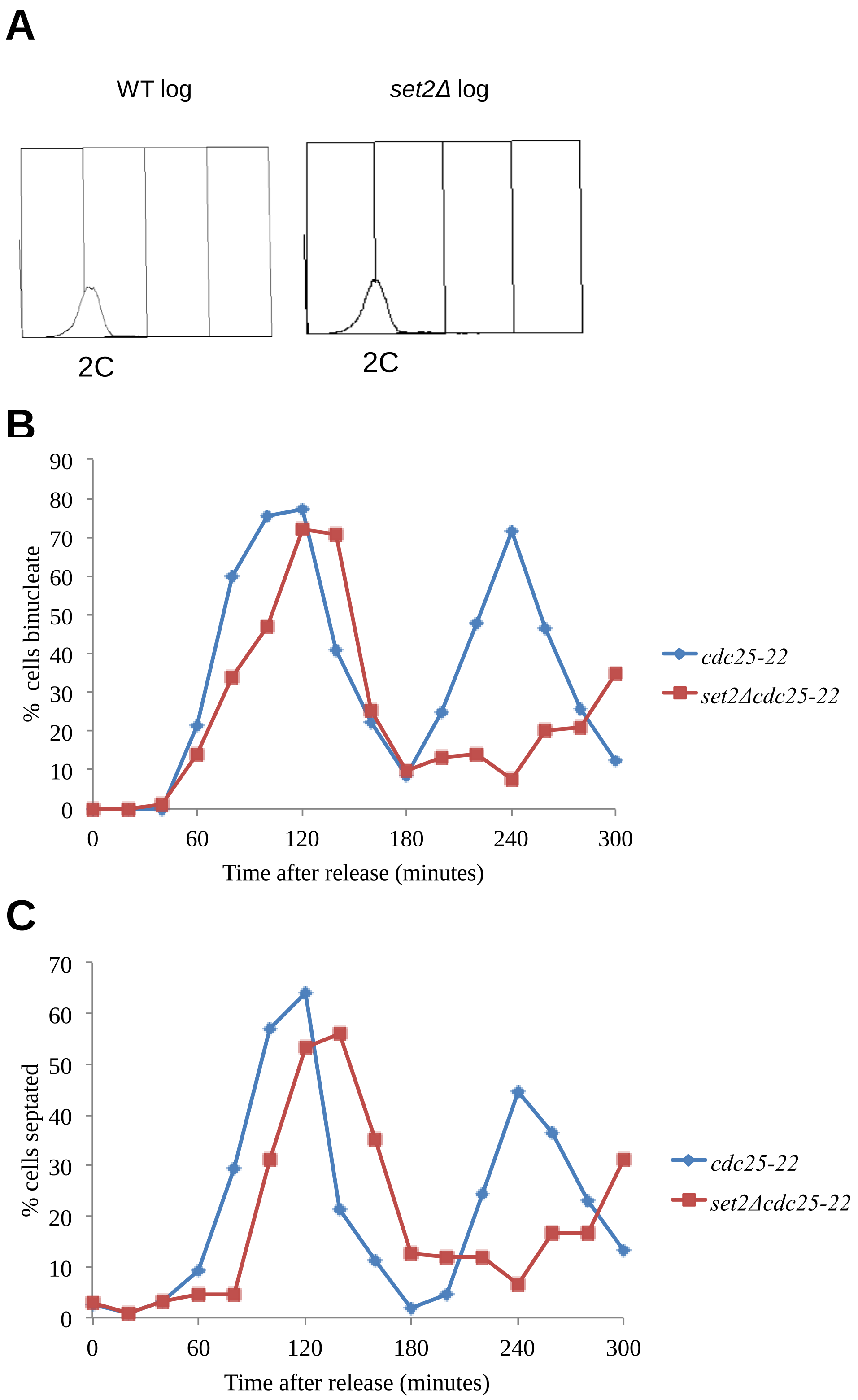

Figure S1 *set2Δ* cells exhibit significant S-phase delay in synchronised cell culture, related to Figure 1. (A) A wild-type or *set2Δ* strain was grown in YES medium to early log phase (OD 0.2-0.5) at 32ºC. Cells from wild-type (WT) or *set2Δ* cells were fixed with 70% ethanol, and examined by flow cytometry determining DNA contents of the cells. (B) Cell cycle analysis of *cdc25-22* and *set2*Δ *cdc25-22* cells following release from *cdc25-22* block (35.5°C; 4.25 hours). Percentage of binucleate cells following release from *cdc25-22* block. (C) Percentage of septated cells following release from *cdc25-22* block.

With normalisation	Subtelomeric number of oris	Top 10 percentage average (%)	Top 100 average (%)	Top 200 average (%)
WT-18℃	40	80.92215	84.38209	76.12337
WT-34℃	38	87.34496	88.9758	81.36208
set2Δ-18℃	39	75.60882	79.5611	70.82381
set2Δ-34℃	41	83.43062	85.93798	77.78305

Without normalisation	Subtelomeric number of oris	Top 10 percentage average (%)	Top 100 average (%)	Top 200 average (%)
WT-18℃	40	35.85002	37.31833	33.72313
WT-34℃	38	57.22798	58.25121	53.18669
set2Δ-18℃	39	33.88988	35.65498	31.76588
set2Δ-34℃	41	56.5458	58.22781	52.72425

Figure S2 Analysis of origins usage at sub-telomeric regions or origin efficiency of the whole genome in vegetative wild-type and *set2Δ* cells at 18°C or 34°C, related to Figure 2.

Pai et al Supplementary Figure 3

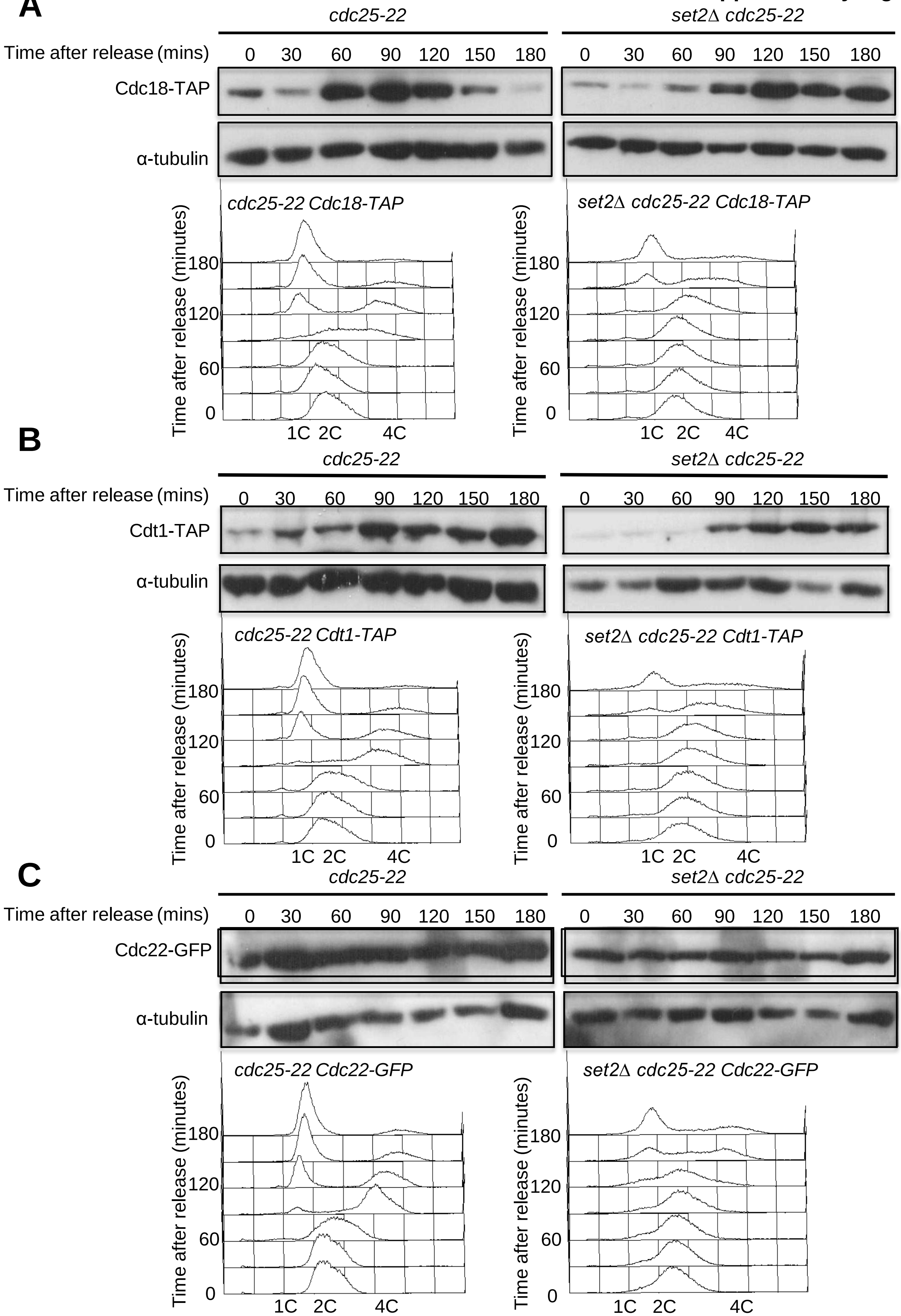

Figure S3 Replication factors are misregulated in *set2*Δ cells, related to Figure 4.(A) Western blot analysis of Cdc18-TAP levels following release from a *cdc25-22* block (35.5°C; 4.25 hours) in *cdc25-22* and *set2*Δ *cdc25-22* cells. FACS analysis of *cdc25-22* and *set2*Δ *cdc25-22* cells following release from a *cdc25-22* block. (B-C) Similar experiments were carried out as described in (A).

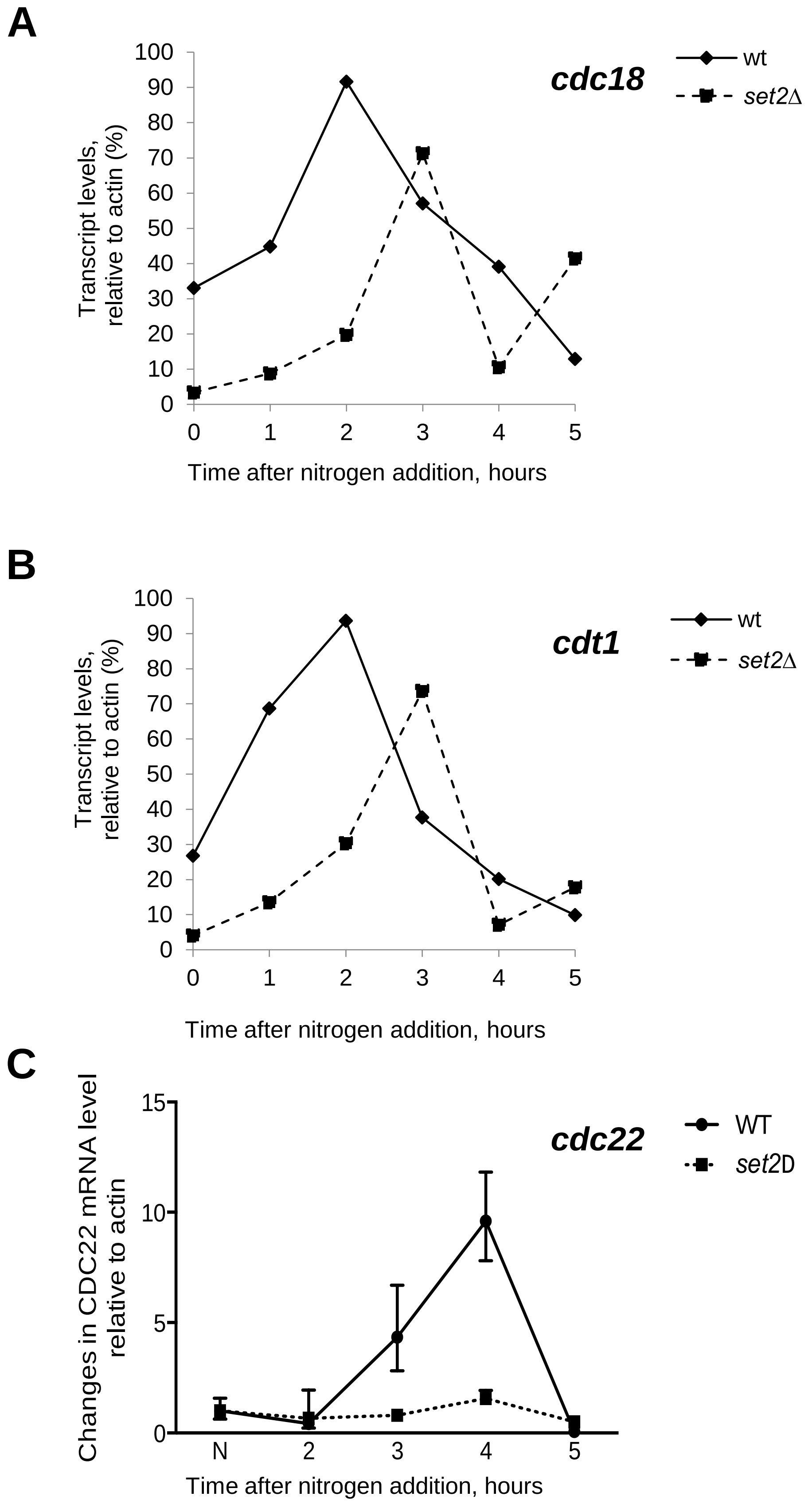

Figure S4 Replication factors are misregulated in *set2*Δ cells, related to Figure 4. Wild-type and *set2*Δ cells were arrested in EMM-N for 16 h at 25°C and then released into EMM+N. Samples for RNA extraction were collected at 0-5 hours after release from G1 arrest. RNA was extracted with Qiagen RNeasy kit and relative transcript levels of *cdc18* (A), *cdt1* (B) *and cdc22* (C) were established by RTqPCR.

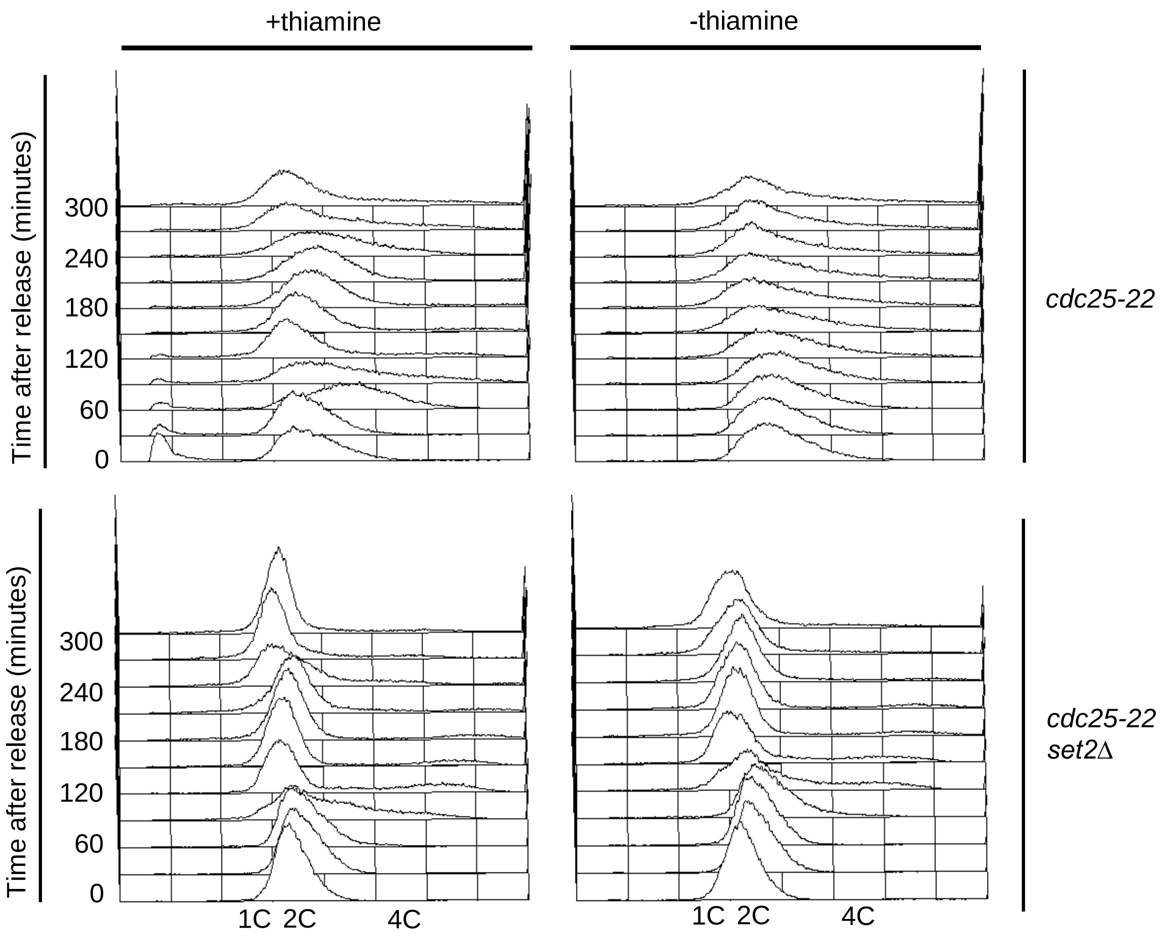

Figure S5 Ectopic expression of *cdc18* and *cdt1* does not rescue the cell cycle defect of *set2*Δ cells, related to Figure 4. *cdc25-22* or *set2*Δ *cdc25-22* cells were grown with (+thiamine) or without (-thiamine for 16 hours) ectopic expression of *cdc18* and *cdt1* from a REP81X plasmid, synchronised at G2/M using the *cdc25-22* block (35.5°C; 4.25 hours) and samples for flow cytometry taken every 30 minutes following release from *cdc25-22* block.

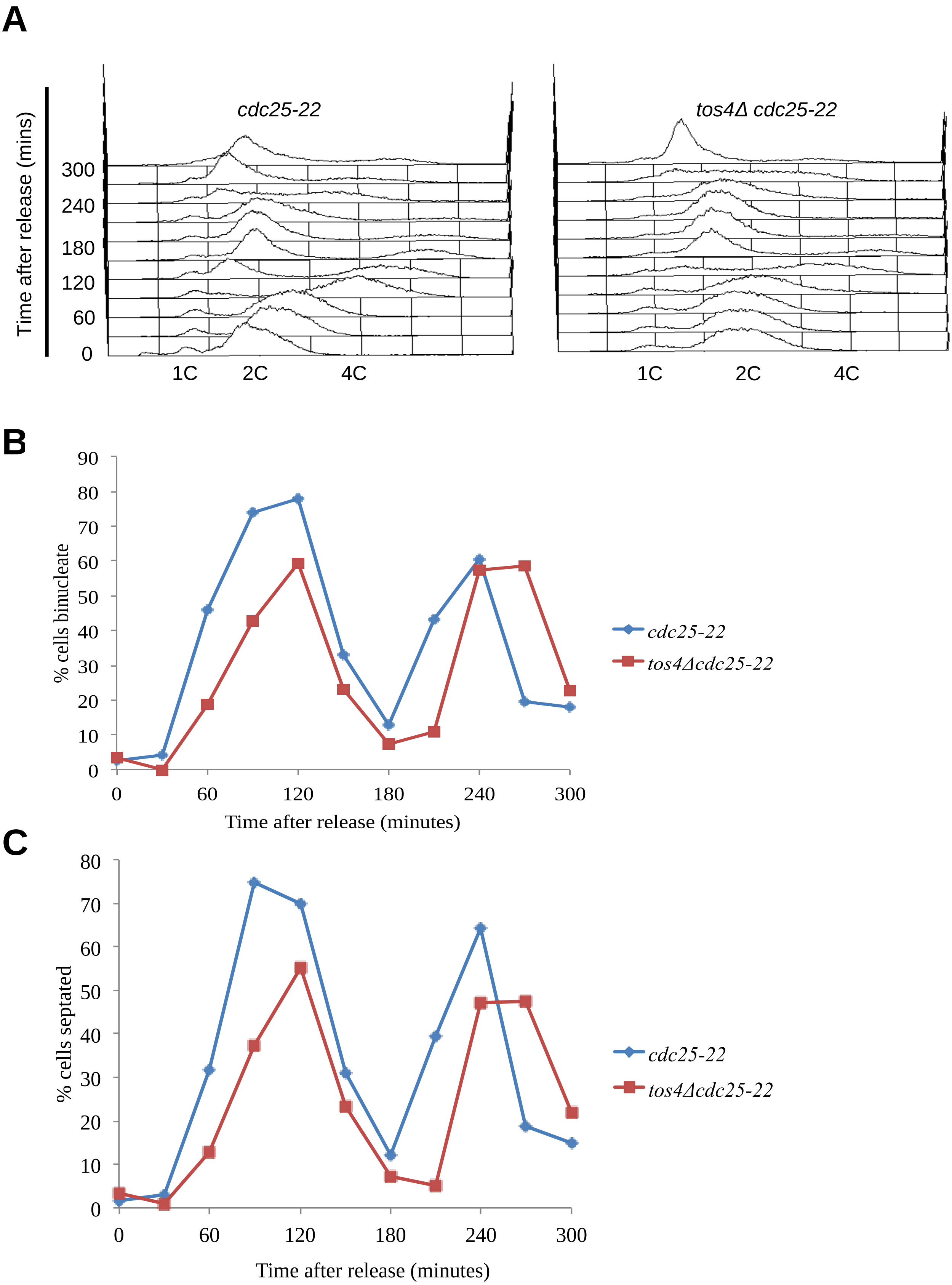

Figure S6 *tos4Δ* cells progress normally through S phase, related to Figure 3. (A) Cell cycle analysis of *cdc25-22* and *tos4Δ cdc25-22* cells following release from *cdc25-22* block (35.5°C; 4.25 hours). (B) Percentage of cells binucleate following release from *cdc25-22* block. (C) Percentage of cells septated following release from *cdc25-22* block.

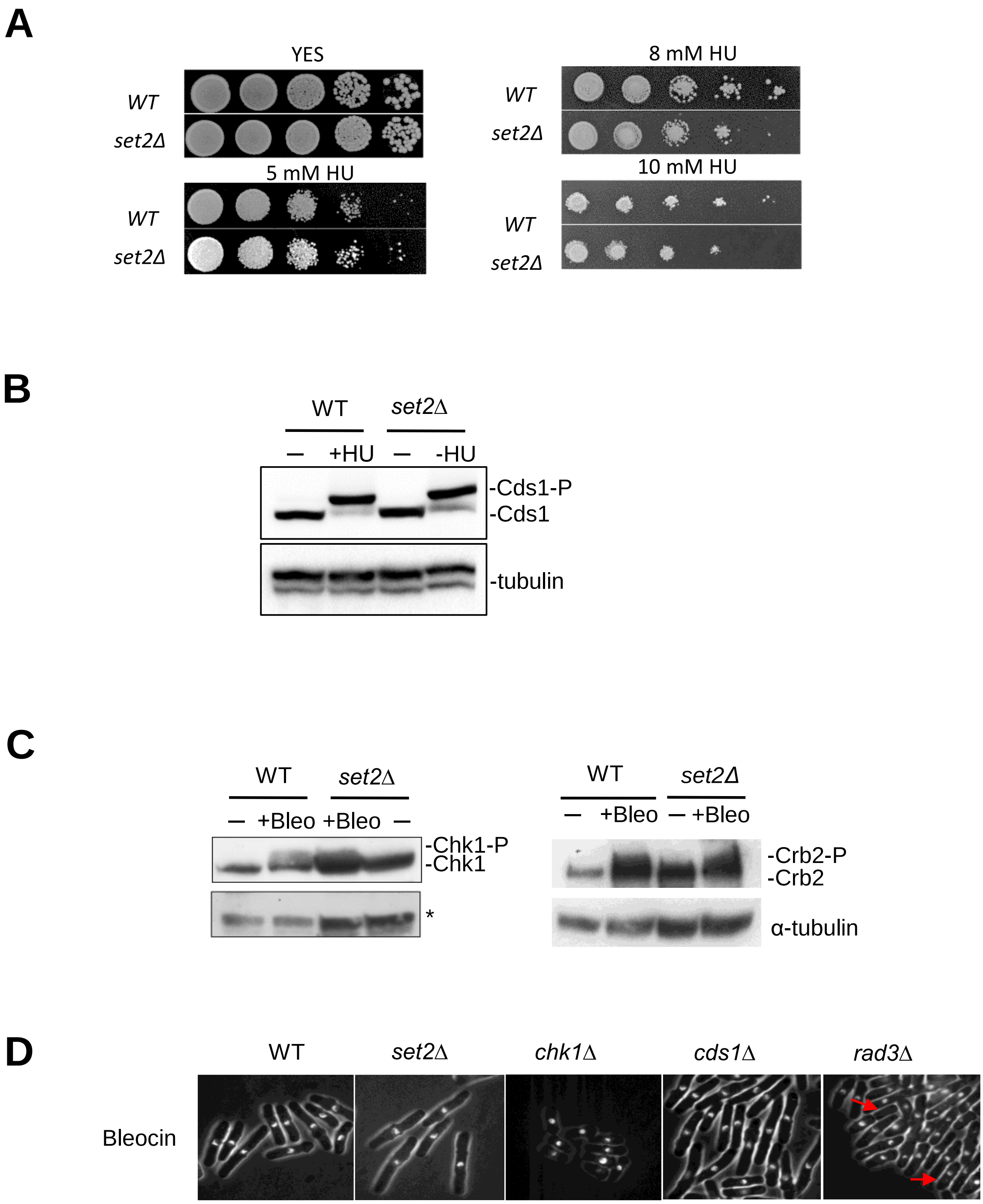

Figure S7 Set2 is not required for checkpoint activation in response to DNA damage and replication stress, related to Figure 6. (A) Serial dilution of a wild-type and *set2Δ* strains were spotted onto YES medium containing different concentration of HU as indicated. Plates were incubated at 30°C for 2-3 days (B) *Cds1-TAP* or *set2Δ Cds1-TAP* cells were grown in YES medium with or without 10 mM HU at 32°C. Western blotting of 2h HU-treated or untreated cell extracts for Cds1-TAP. α-tubulin was used as a loading control. (C) Western analysis of Chk1 or Crb2 phosphorylation following 1h treatment with 5µg/ml bleomycin at 26°C in wild-type or *set2Δ* cells. Asterisk indicates unspecific bands. (D) Each fixed culture from WT, *set2Δ chk1Δ*, *cds1Δ*, or *rad3Δ* strain was stained with DAPI and analysed by fluorescence microscopy showing nuclear morphology for each strain. Red arrows indicate the cut phenotype in the samples collected after 2h Bleocin treatment at 26°C.

Table S1, related to Figure 1-7. Strains used in this study

Strain	Genotype	Source
TH 451	*chk1::ura4 ade- leu1-32 h+*	Lab stock
TH 758	*cds1::ura4 ura4-D18 leu1-32 h-*	Lab stock
TH 1543	*cdc25-22 leu1-32 ade6-D1, ura4-D18 his3D h+*	Lab stock
TH 2094	*arg3-D4, ade6-D1, ura4-D18, leu1-32, his3-D1 h-*	Lab stock
TH 3645	*spd1::hygroR ura4-D18 leu1 ade6-704 h-*	Olaf Nielsen
TH 3271	*set2::ura4+ ade6-210 arg3-D4 his3-D1 leu1-32 ura4-D18 h-*	Robin Allshire
TH 6236	*set2::kanMX ade6-210 leu1-32 ura4-D18 h-*	Robin Allshire
TH 6237	*set2::kanMX ade6-210 leu1-32 ura4-D18 h+*	Robin Allshire
TH 6707	*set2::ura4 cdc25-22*	This study
TH 6877	*972 h-*	Lab stock
TH 6932	*tos4::KanMX cdc25-22*	This study
TH 6919	*set2::kanMX*	Robin Allshire
TH 6999	*set2::ura4 cdc25-22, pREP81x-cdt1 pREP81x-cdc18*	This study
TH 7002	*cdc25-22 leu1-32 ade6-D1, ura4-D18 his3D pREP81x-cdt1 pREP81x-cdc18*	This study
TH 7133	*set2::set2-R255G-ura4*	Lab stock
TH 7952	*set2::kanMX chk1::ura4*	This study
TH 8459	*set2::kanMX spd1::HygroR*	This study
TH 8590	*crb2-YFP-leu+ crb2::ura4 set2::kanMx*	This study
TH 8658	*cds1::kanMX set2::kanMX*	This study
TH 8659	*rad3::kanMX set2::kanMX*	This study
TH 8664	*set2::kanMX yox1:: kanMX*	This study
TH 8662	*cdc18-TAP::kanMX*	Stephen Kearsey
TH 8669	*set2:: kanMX cdc18-TAP::kanMX*	This study
TH 8672	*cdt1-TAP-KanMX*	Stephen Kearsey
TH 8726	*set2::kanMX cdc22-D57N*	This study
TH 8727	*cdc22-CFP:: kanMX*	Stephen Kearsey
TH 8729	*set2:: kanMX cdc22-CFP:: kanMX*	This study
IM 655	*rnh201::KanMX cdc20-M603F::lox ade6-704 leu1-32 ura4-D18 h-*	Tony Carr

IM 856	*rnh201::KanMX cdc6-L591G:lox ade6-704 leu1-32 ura4-D18 h-*	Tony Carr
YAK250	*set2::kanMx rnh201::HYG, cdc20-M603F ade6-704 leu1-32 ura4-D18 h-*	Tony Carr
YAK251	*set2::kanMx rnh201::HYG, cdc6-L591G ade6-704 leu1-32 ura4-D18*	Tony Carr
RBP43	*Res1-13myc:: kanMX, leu1-32, ura4-D18*	Rob de Bruin
RBP751	*Res1-13myc:: kanMX, set2::ura4, ade6-210, arg3-D4, his3-D1, leu1-32 ura4-D18*	Rob de Bruin
MG83	*ura4::adh-dmNK-NAT-adh-hENT1 ura4-aim*	Edgar Hartsuiker

Table S2, related to Figure 3, Figure 4 and Figure 7. Primers used in this study

		FW	RV
cdc18	RTqPCR	GTAGGCATGCAATTGAACTTGCGG	TCATAGCAGATGTCGCTCGGACAA
cdt1	RTqPCR	ACCGTATGGCCAGAGTCATTTGCT	AATTCAATGGAGCGGGAGAAGGCT
cdc22	RTqPCR	TGCAACGTGTTGAACGTAACGAGC	AGGTAATGAACGACGACCACGGTT
tos4	RTqPCR	TTCTGCAGTGAGAAGAGAGCCACT	AACCGTGGATAGGACATGGTCACA
nrm1	RTqPCR	GGGAAAGGCCAACAAACGAAGTGT	ATCGAACCGCAATCGGTGAAATCG
act1	RTqPCR	CGCCGAACGTGAAATTGTTCGTGA	TCAAGGGAGGAAGATTGAGCAGCA
cdc18	ChIP qPCR	GGCATTTCATATCTTTGAGGATGAGTCGT	ATGTCGCGTTCAACTCTACGTGTC
cdt1	ChIP qPCR	TTTCAGAGAGCCTGAACTTGG	CTCCTTTGCTCTGCGAGATATTA
cdc22	ChIP qPCR	ACTTAAAGTTCGGATGACGCGACG	GTTTGTAAGGTGGTAAATACCGGG
tos4	ChIP qPCR	CACTGGGTTACTCTCGTTTCTT	CCTGGGTATAAACACGCTATGA
byr3	ChIP qPCR	TGGCAAGTTGTTGTGCTTCTTCCG	TAACAAGCACATGGTGGCACTTGG
rps17	ChIP qPCR	GCACCTGGTTTGTTGTTGGTTG	TTCGTAACCTCCGTCGCTTCTGTT
